# Antiviral Protection via RdRP-Mediated Stable Activation of Innate Immunity

**DOI:** 10.1371/journal.ppat.1005311

**Published:** 2015-12-03

**Authors:** Meghan M. Painter, James H. Morrison, Laurie J. Zoecklein, Tommy A. Rinkoski, Jens O. Watzlawik, Louisa M. Papke, Arthur E. Warrington, Allan J. Bieber, William E. Matchett, Kari L. Turkowski, Eric M. Poeschla, Moses Rodriguez

**Affiliations:** 1 Mayo Graduate School of Medicine, Mayo Clinic, Rochester, Minnesota, United States of America; 2 Department of Molecular Medicine, University of Colorado School of Medicine, Aurora, Colorado, United States of America; 3 Department of Medicine, University of Colorado School of Medicine, Aurora, Colorado, United States of America; 4 Division of Infectious Diseases, University of Colorado School of Medicine, Aurora, Colorado, United States of America; 5 Department of Neurology, Mayo Clinic, Rochester, Minnesota, United States of America; 6 Department of Neurosurgery, Mayo Clinic, Rochester, Minnesota, United State of America; 7 Department of Immunology, Mayo Clinic, Rochester, Minnesota, United States of America; University of Pennsylvania School of Medicine, UNITED STATES

## Abstract

For many emerging and re-emerging infectious diseases, definitive solutions via sterilizing adaptive immunity may require years or decades to develop, if they are even possible. The innate immune system offers alternative mechanisms that do not require antigen-specific recognition or *a priori* knowledge of the causative agent. However, it is unclear whether effective stable innate immune system activation can be achieved without triggering harmful autoimmunity or other chronic inflammatory sequelae. Here, we show that transgenic expression of a picornavirus RNA-dependent RNA polymerase (RdRP), in the absence of other viral proteins, can profoundly reconfigure mammalian innate antiviral immunity by exposing the normally membrane-sequestered RdRP activity to sustained innate immune detection. RdRP-transgenic mice have life-long, quantitatively dramatic upregulation of 80 interferon-stimulated genes (ISGs) and show profound resistance to normally lethal viral challenge. Multiple crosses with defined knockout mice (*Rag1*, *Mda5*, *Mavs*, *Ifnar1*, *Ifngr1*, and *Tlr3)* established that the mechanism operates via MDA5 and MAVS and is fully independent of the adaptive immune system. Human cell models recapitulated the key features with striking fidelity, with the RdRP inducing an analogous ISG network and a strict block to HIV-1 infection. This RdRP-mediated antiviral mechanism does not depend on secondary structure within the RdRP mRNA but operates at the protein level and requires RdRP catalysis. Importantly, despite lifelong massive ISG elevations, RdRP mice are entirely healthy, with normal longevity. Our data reveal that a powerfully augmented MDA5-mediated activation state can be a well-tolerated mammalian innate immune system configuration. These results provide a foundation for augmenting innate immunity to achieve broad-spectrum antiviral protection.

## Author Summary

We show in transgenic mouse and human cell culture models that expression of a viral RNA-dependent RNA polymerase (RdRP) profoundly reconfigures mammalian innate antiviral immunity by exposing the normally stringently-sequestered viral RNA copying process to MDA5 detection. In RdRP-transgenic mouse tissues, the mRNAs for critical interferon-stimulated genes (ISGs) are highly elevated, up to 300-fold. Many antiviral ISGs are similarly induced, up to 560-fold, when we express the RdRP in human monocyte and lung epithelial cell lines. The elevated basal ISG profile protects RdRP mice and human cells from viral infection. RdRP mice are robustly healthy with normal longevity despite life-long, constitutive MDA5-mediated innate immune system activation. This work suggests a plausible approach to augmenting innate immunity to block viral pathogenesis.

## Introduction

Most viruses store and replicate their genetic information as RNA, with no DNA component [1]. RNA virus pathogens include flaviviruses (e.g., dengue, West Nile, hepatitis C), picornaviruses (polio, rhino, Coxsackie, hepatitis A, foot and mouth, enterovirus D68), coronaviruses (SARS, MERS), calciviruses (Norwalk), togaviruses (Chikungunya), filoviruses (Ebola, Marburg), paramyxoviruses (Nipah, Hendra, measles), rhabdoviruses (rabies), arenaviruses (Lassa), orthomyxoviruses (influenza A), and bunyaviruses (hanta, Crimean-Congo), and many lack effective vaccines or therapeutic strategies. All RNA viruses encode RNA-dependent RNA polymerases (RdRPs), which replicate the viral RNA genome and transcribe it into mRNA in a way that requires generation of double-stranded RNA (dsRNA) intermediates.

Whereas plants and worms encode RdRPs as components of RNA silencing systems, vertebrates do not [1]. Thus, mammalian innate immune systems sense the replication intermediates synthesized by these viral replicases as pathogen-associated molecular patterns (PAMPs). In mammals, the resulting activation of cell-intrinsic innate immunity culminates in production of type I interferons (IFNs) and activation of a large class of potent antiviral factors collectively termed IFN-stimulated genes (ISGs) [2].

RNA viruses have evolved mechanisms to isolate RdRP activity during viral replication. Positive-strand RNA viruses sequester the entire replication machinery to ultrastructurally-distinctive membrane-bound cytoplasmic compartments (termed replication factories) that they generate by massively re-engineering host organelle membranes [3]. For example, dengue virus replication occurs inside elaborately convoluted vesicles that contain viral dsRNA. High-resolution electron microscope tomography shows a continuous endoplasmic reticulum-derived membranous network with small pores through which plus-strand progeny genomes are released to the cytoplasm [4]. The RdRP of poliovirus, a picornavirus, is similarly associated with complex virus-engineered membranous structures although the precise morphology and topology, with respect to the cellular milieu, are unclear. Immuno-electron microscopic labeling has suggested that the poliovirus replication complex localizes to the cytoplasmic face of compact membrane vesicles that are, in turn, surrounded by larger elongated (rosette-like) vesicular structures [5,6,7].

The universality of strict membrane sequestration of the viral replication complex among the large and very diverse group of positive-strand RNA viruses is compelling, but there is limited understanding of this structure’s function; it is thought that these membranes concentrate and provide physical support for the viral synthetic apparatus. However, by sequestering the RdRP and its viral dsRNA intermediates from the cytosol, they might also prevent detection by the innate immune system [3].

In plants, transgenic expression of viral polymerases can confer resistance to viral infection [8,9,10,11,12,13], but a clear mechanism remains to be defined [14]. In cultured vertebrate cell lines, parainfluenza virus RdRP [15] or Semliki Forest virus RdRP [16] expression induce IFN-β, albeit by different mechanisms. We reported that mice made transgenic for a picornaviral RdRP—the 3D^pol^ protein of Theiler’s murine encephalomyelitis virus (TMEV)–resist infection by RNA and DNA viruses [17]. Here we determined the mechanism for this profound effect. In the mouse and in human cell lines, we show that ectopic expression of a catalytically-active RdRP produces dsRNA, a potent MDA5 agonist, which leads to constitutive ISG signaling and renders RdRP-expressing cells refractory to viral infection.

Importantly, in RdRP mice, this highly amplified MDA5-mediated innate immune state persists life-long, yet the animals have normal health and longevity. This unprecedented result contrasts markedly with naturally-occurring human genetic diseases or analogous transgenically-engineered mouse models, in which constitutive MDA5 activation results in severe autoimmune diseases such as systemic lupus erythematosus (SLE) or Acardi-Goutieres syndrome (AGS) [18,19]. Our data indicate that ectopic expression of a viral RdRP stimulates innate immunity, and that mammalian signaling pathways exist that permit lifelong MDA5-mediated ISG activation without inflammatory disease.

## Results

### RdRP mice constitutively upregulate antiviral effectors

**Fig 1:**
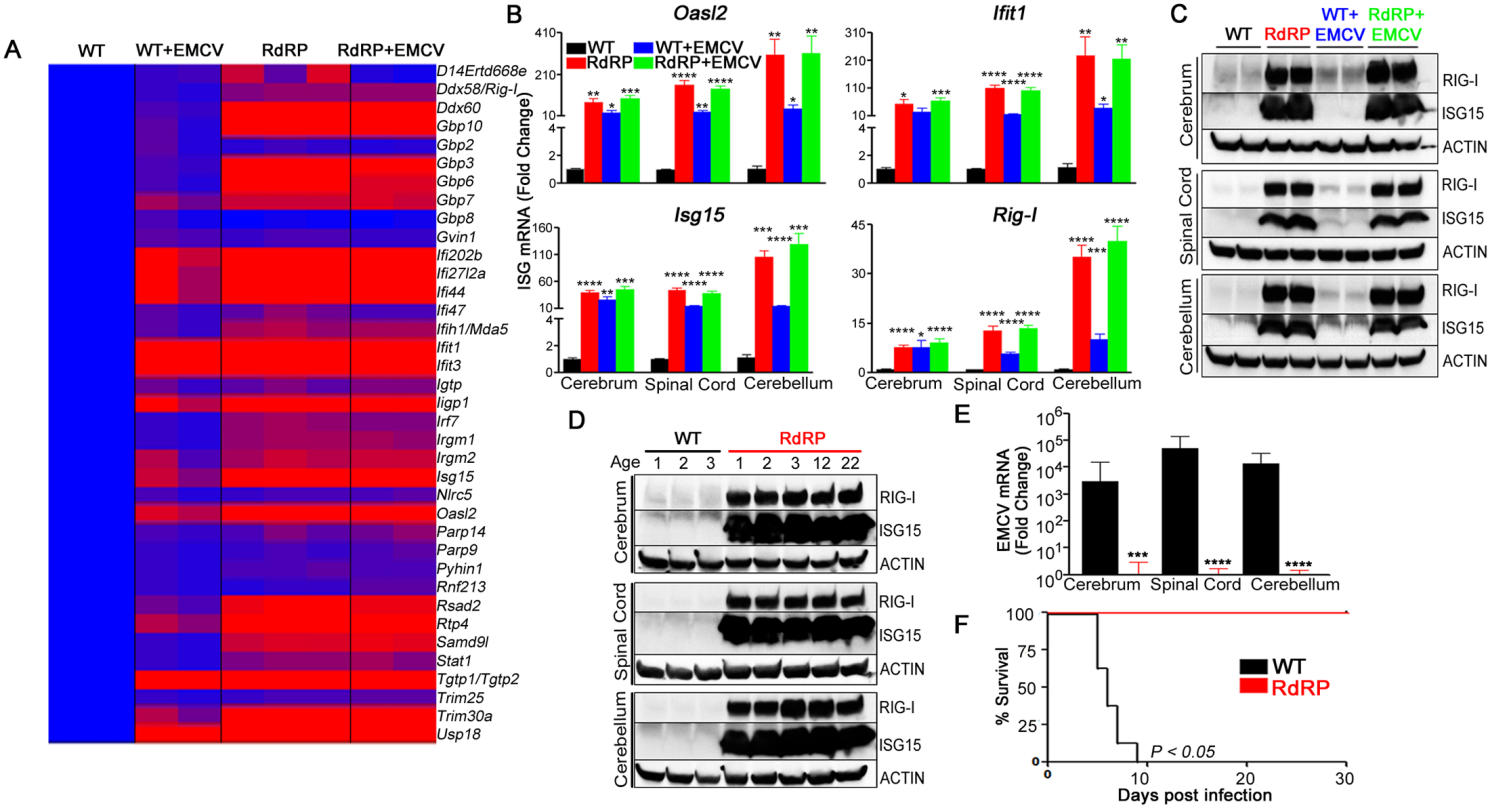
RdRP mice display highly augmented antiviral defenses. (A) Heat map of differentially expressed genes in CNS tissue. Microarray data are presented as fold change (blue = 1-fold induction; purple = 8-fold upregulation; red = ≥15-fold upregulation). WT+EMCV: expression profile of genes significantly upregulated >4-fold in spinal cords of WT FVB mice infected with EMCV (n = 2) compared with uninfected WT FVB mice (n = 2). RdRP: uninfected transgenic RdRP FVB mice (n = 3) compared with uninfected WT FVB mice (n = 3), data are representative of two independent experiments. RdRP+EMCV: RdRP FVB mice infected with EMCV (n = 2) compared with uninfected WT FVB mice (n = 2). For a complete list of upregulated genes see S1–S3 Tables. (B) mRNA levels of the prominent antiviral genes, *Oasl2*, *Isg15*, *Ifit1*, and *Rig-I*, determined by RT-PCR in cerebrum, spinal cord, and cerebellum tissues of uninfected (WT FVB or RdRP FVB) or virally-infected FVB mice (WT+EMCV or RdRP+EMCV) (n = 5 mice per group, relative to *Gapdh* mRNA, mean ± SEM). (C and D) Cerebrum, spinal cord, and cerebellum tissue homogenates (C, n = 2 mice per group, 1 per lane; D, n = 1 uninfected mouse of specified age in months per lane) were analyzed by immunoblotting using antibodies for RIG-I and ISG15. Antibody for β-ACTIN served as control. (E) RT-PCR analysis of viral titers in murine tissues two days post infection with EMCV (n = 8 mice per genotype, relative to endogenous *Gapdh* mRNA, mean ± SEM). (F) Survival curve following EMCV infection (n = 8 mice per genotype). Results are presented as a Kaplan-Meier plot and are representative of two independent experiments. * *P<0*.05; ** P<0.01; *** P<0.001; **** P<0.*0001*.

We first determined gene expression profiles in tissues of wild-type (WT) and RdRP-transgenic mice, before and after intraperitonal infection with a lethal dose of encephalomyocarditis virus (EMCV). Whole-genome mRNA microarray analyses of spinal cords from uninfected WT mice and EMCV-infected WT mice revealed that 37 genes (fold change criterion > 4.0 and *P <* 0.05) were upregulated significantly, up to 26.5-fold, in EMCV-infected mice compared to uninfected WT controls (Fig 1A WT+EMCV, S1A Fig, S1 Table). In striking contrast, in spinal cords of uninfected RdRP mice, 80 gene mRNAs were elevated significantly. In addition, this baseline upregulation was quantitatively dramatic, up to 114-fold compared to uninfected WT controls (Fig 1A RdRP, S1A Fig, S2 Table), and of the 80 elevated genes, 30 (38%) were elevated more than 10-fold. Importantly, of the 37 genes upregulated following lethal viral infection in WT mice, 97% were intrinsically upregulated > 4-fold in uninfected RdRP mice. In spinal cords from EMCV-infected RdRP mice, 79 genes were significantly upregulated, up to 124-fold, compared with uninfected WT controls (Fig 1A RdRP+EMCV, S1A Fig, S3 Table). Moreover, there was no statistically significant difference between the gene patterns of uninfected RdRP and virus-infected RdRP mice. These results demonstrate that RdRP mice possess, at baseline, a maximally upregulated innate immune transcriptome which is capable of effecting a robust antiviral response. In both breadth and extent, it far exceeds the innate immune gene inductions produced by virus infection itself (80 vs. 37 genes, mean fold induction 13.9-fold in uninfected RdRP vs. 8.5-fold in EMCV-infected WT).

Since *Oasl2*, *Ifit1*, *Isg15*, and *Rig-I*, were highly upregulated (114, 74, 40, and 10-fold, respectively) in neural tissue of uninfected RdRP mice, we used these genes as a representative cohort of antiviral genes to verify microarray results and to determine whether antiviral effectors were upregulated in other tissues of uninfected RdRP mice. RT-PCR revealed systemic upregulation of antiviral genes (up to 300-fold) in diverse RdRP murine tissues, including the CNS (Fig 1B), lung, kidney, heart, and liver (S1B Fig). Stable, life-long increased protein levels were present in CNS tissues of RdRP mice irrespective of virus infection status (Fig 1C and 1D), and this elevated ISG profile was not cell-type specific (S1C Fig).

Next, we investigated whether this inherent upregulation of antiviral effectors contributes to decreased viral burden in tissues of infected RdRP animals. Mice were challenged intraperitoneally with a lethal dose of EMCV and viral titers were assessed two days later in target tissues. In the cerebrum, spinal cord, and cerebellum, RdRP mice exhibited dramatically decreased viral titers compared to WT controls (Fig 1E). Most importantly, EMCV was fatal in WT mice, whereas RdRP mice were resistant (Fig 1F). Comparison of RdRP mice with control transgenic mice that express viral protein 1 (VP1) or viral protein 2 (VP2) of TMEV showed that generation of an antiviral phenotype is specific to RdRP expression (S1D–S1G Fig).

### RdRP-mediated antiviral effects operate independently of the adaptive immune system

**Fig 2:**
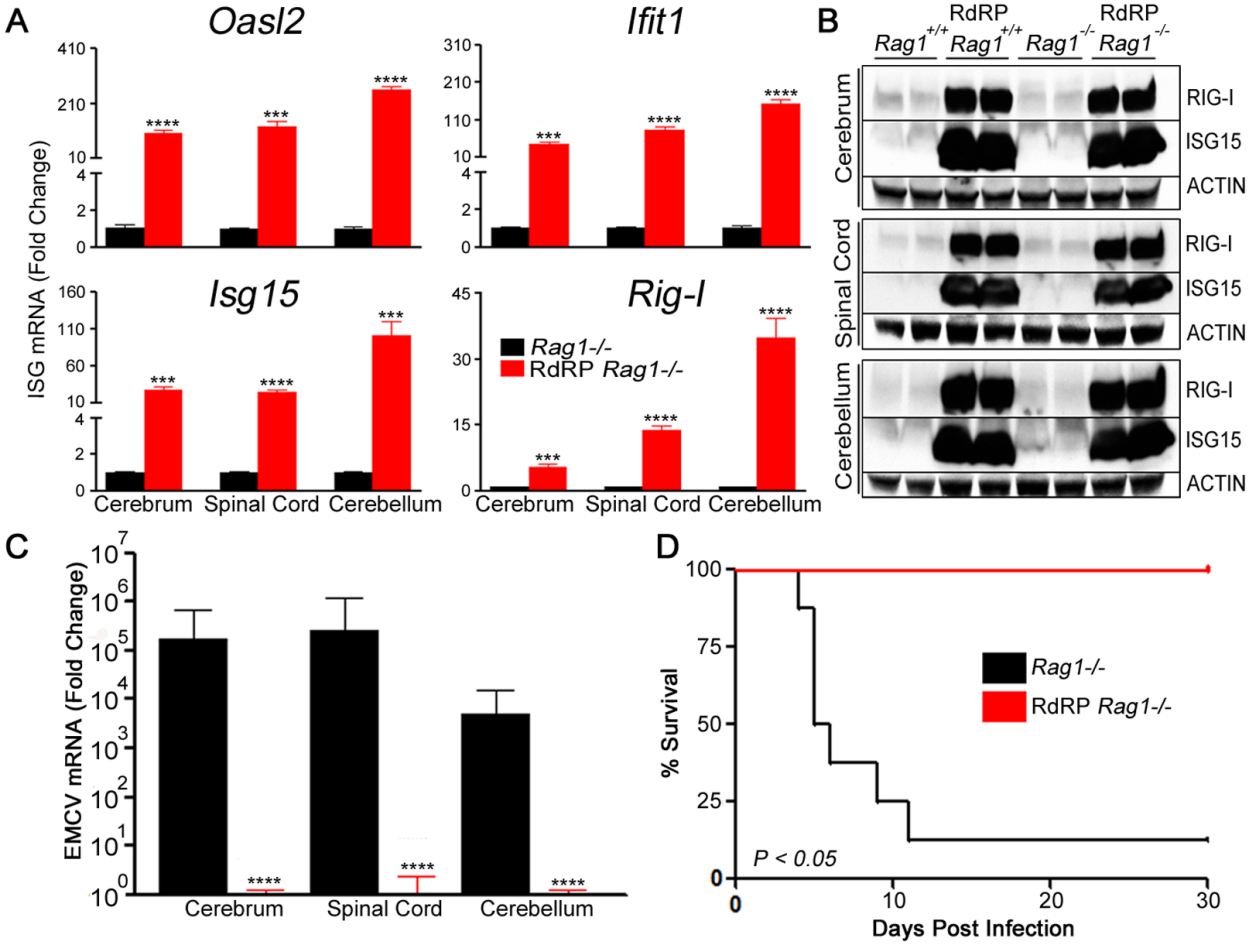
RdRP-induced antiviral phenotype acts independently of intact adaptive immune signaling. (A) RT-PCR analysis of *Oasl2*, *Isg15*, *Ifit1*, and *Rig-I* mRNA levels in cerebrum, spinal cord, and cerebellum of uninfected FVB mice (n = 5 per genotype, relative to *Gapdh* mRNA, mean ± SEM). (B) Cerebrum, spinal cord, and cerebellum tissue homogenates (n = 2 uninfected FVB mice per genotype, 1 per lane) were analyzed by immunoblotting using antibodies for RIG-I and ISG15. Antibody for β-ACTIN served as control. (C) RT-PCR analysis of viral titers in murine tissues two days post infection with EMCV (n = 8 mice per genotype, relative to endogenous *Gapdh* mRNA, mean ± SEM). (D) Survival curves (Kaplan-Meier plot) following EMCV infection (n = 8 mice per genotype). Data are representative of two independent experiments. *** P<0.001; **** P<0.*0001*.

A key question was whether maintenance of RdRP-induced antiviral effects depends on an intact adaptive immune system. We previously crossed TMEV RdRP-transgenic mice with *Rag1*-deficient mice, generating RdRP mice with complete absence of mature T and B cells, and observed that these mice have enhanced survival after TMEV challenge [20]. Here we carried out gene expression analyses and heterologous viral challenges with EMCV to determine the mechanism; RdRP-*Rag1* ^*-/-*^ mice maintain constitutive upregulation of antiviral ISGs (Fig 2A and 2B), exhibit up to 5.5-logs reduced viral transcript loads (Fig 2C), and survive lethal EMCV challenge (Fig 2D). These results show that RdRP-induced intrinsic upregulation of critical innate immune effectors is sufficient to confer marked resistance to viral infection.

### Requirement for MDA5, MAVS, and IFNαβR

To further characterize the molecular mechanism by which the RdRP transgene upregulates protective antiviral effectors *in vivo*, we focused on key steps in classic innate antiviral sensing. Viral molecular and structural motifs are detected by the cell-intrinsic innate immune system using pattern recognition receptors (PRRs). TLR3 is a PRR localized to the endosomal compartments, and RIG-I, MDA5, and LGP2 are RIG-I-like receptor RNA helicases with essential roles in cytosolic viral sensing [21,22]. Recognition of viral motifs activates receptor-proximal signaling molecules, namely TRIF in the TLR3 pathway and MAVS in the RIG-I/MDA5 pathway, which converge on phosphorylation of members of the IFN regulatory factor family and production of type I IFNs. IFNα or IFNβ engagement with the cognate receptor (IFNαβR) on the plasma membrane recruits transcription factors which bind to IFN-stimulated response elements (ISREs) to upregulate ISGs [2]. IFN-stimulated effectors can also be activated through the IFNγR and transcription factor binding to IFNγ-activated site (GAS) promoter elements. Normally, these responses are highly constrained temporally, with acute sensor activation and ISG upregulation after the onset of viral infection, followed by resolution to baseline as infection is controlled by innate and adaptive immune responses.

**Fig 3:**
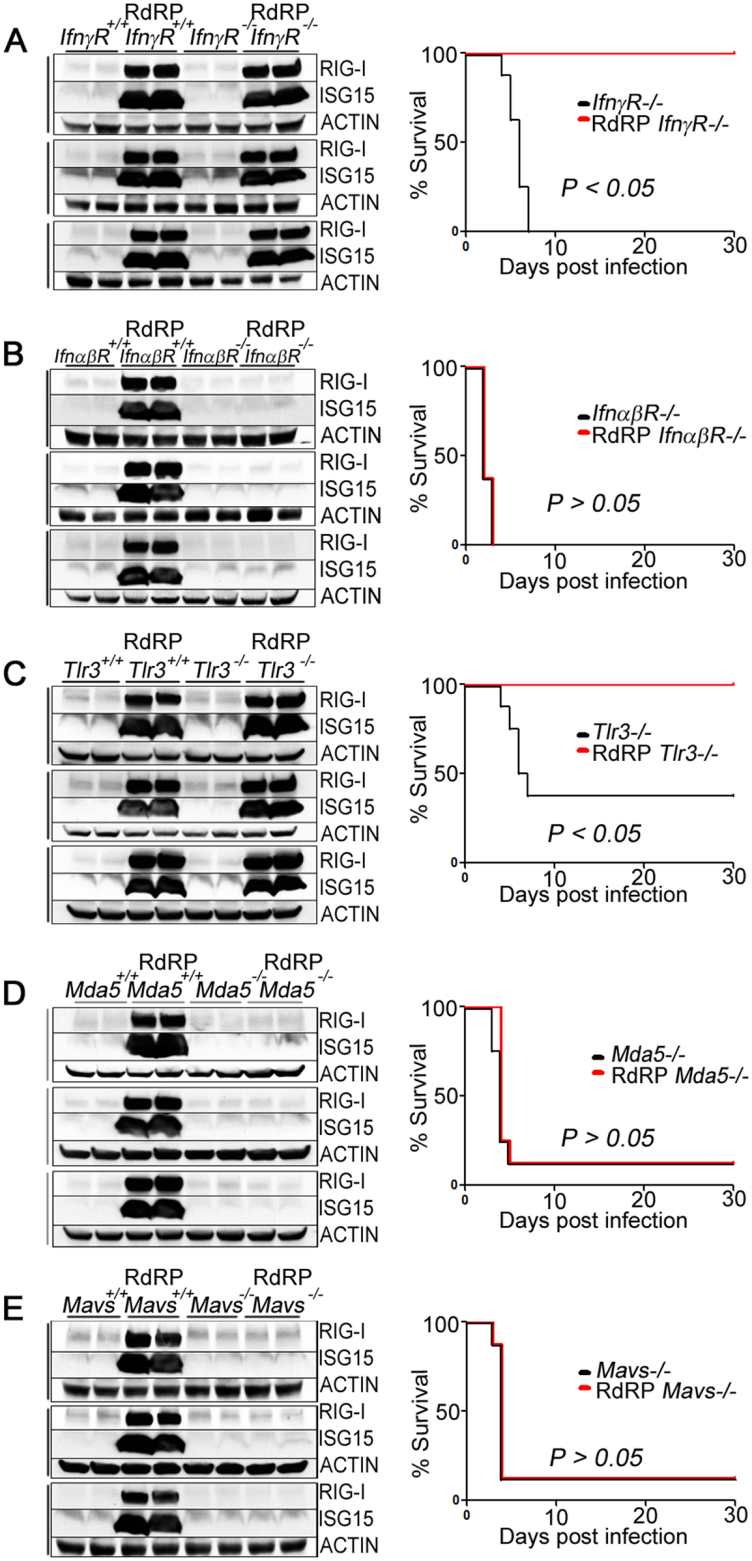
RdRP-induced antiviral phenotype is mediated in a MDA5-MAVS, IFNαβR-dependent manner. (A-E) Knockout mice were bred to RdRP mice and the antiviral phenotype of each cross was analyzed by immunoblotting and survival. Left, cerebrum (top panel), spinal cord (middle), and cerebellum (bottom) tissue homogenates (n = 2 uninfected mice per genotype, 1 per lane) were analyzed by immunoblotting. Right, survival curve following EMCV infection (n = 8 mice per genotype), data are representative of two independent experiments. (A) *IfnγR* ^*-/-*^ cross. (B) *IfnαβR* ^*-/-*^ cross. (C) *Tlr3* ^*-/-*^ cross. (D) *Mda5* ^*-/-*^ cross. (E) *Mavs* ^*-/-*^ cross.

We first tested the IFN-dependence of this protective pathway by crossing RdRP mice to IFN receptor mouse knockout strains (*IfnγR* and *IfnαβR*). The antiviral phenotype of each cross was characterized by determining basal levels of antiviral gene products (RT-PCR and immunoblotting) and survival after viral challenge. The RdRP-*IfnγR* ^*-/-*^ data demonstrated that this cellular receptor is not necessary for the RdRP-induced antiviral effects since RdRP-*IfnγR* ^*-/-*^ mice still highly upregulated antiviral factors and survived lethal viral challenge (Fig 3A and S2A Fig). In clear contrast, the RdRP*-IfnαβR* ^-/-^ mice we generated established that the antiviral phenotype requires intact IFNαβR-mediated signaling; RdRP*-IfnαβR* ^-/-^ mice did not upregulate antiviral factors and succumbed to viral infection similarly to control mice (Fig 3B and S2B Fig).

To determine how RdRP-induced activation of host PRRs triggers constitutive innate antiviral signaling, we crossed RdRP mice to additional mouse knockout strains (*Tlr3*, *Mda5*, and *Mavs*). RdRP-*Tlr3* ^*-/-*^ data demonstrated that this cellular receptor is not necessary for the RdRP-induced antiviral effects since RdRP-*Tlr3* ^*-/-*^ mice still highly upregulated antiviral factors and survived lethal viral challenge (Fig 3C and S2C Fig). However, RdRP-*Mda5* ^*-/-*^ and RdRP-*Mavs* ^*-/-*^ mice did not upregulate antiviral factors and they succumbed to viral infection similarly to control mice (Fig 3D and 3E, S2D and S2E Fig). These experiments identify the MDA5-MAVS nucleic acid sensing pathway as essential.

### RdRP-mediated constitutive activation of the MDA5-MAVS pathway does not cause autoimmunity or other inflammatory pathology

Heterozygous MDA5 missense mutations are the molecular basis of previously genetically uncharacterized cases of the human type I interferonopathy AGS [18]. Furthermore, constitutive activation of the MDA5-MAVS pathway by a gain-of-function mutation in MDA5 (G821S; *Ifih1* ^*gs/+*^) causes a lupus-like autoimmune disease in mice [19]; MDA5 G821S mice display retarded growth, multi-organ pathology, and the majority die within six months of birth. These chronic human and mouse MDA5-dependent diseases are both characterized by marked ISG upregulation.

**Fig 4:**
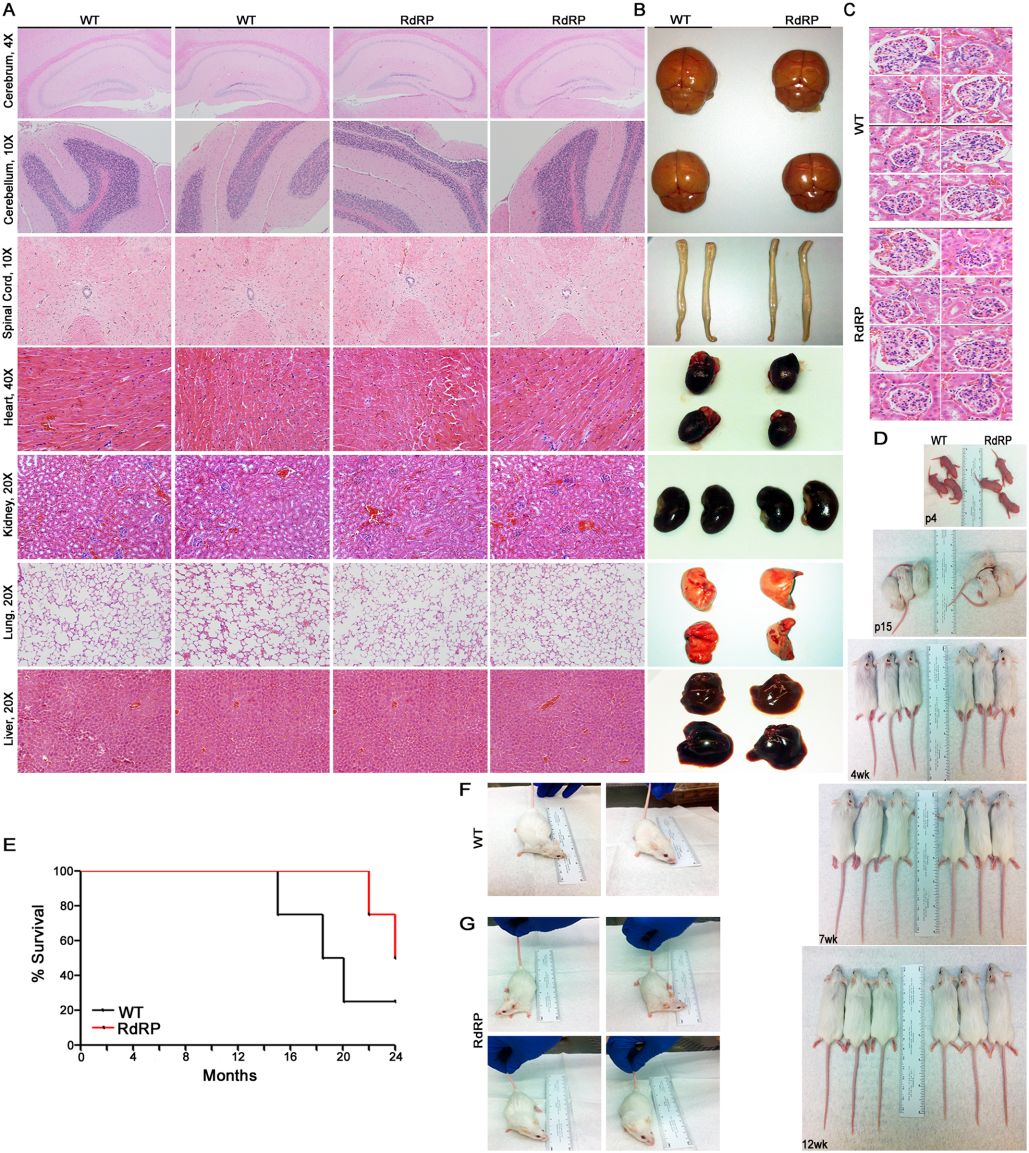
RdRP mice appear indistinguishable from WT controls. (A) Histological examination of WT and RdRP transgenic mice. Tissues were harvested from uninfected age-matched, sex-matched adult FVB mice (n = 2 per genotype). Histological examination was performed on formalin-fixed paraffin-embedded tissue after hematoxylin and eosin staining. (B) Gross tissue morphology. Tissues were harvested from uninfected age-matched, sex-matched adult FVB mice (n = 2 mice per genotype). (C) Kidney tissues were closely examined (40X) for signs of glomerulonephritis. (D) Sex-matched WT (n = 3) and RdRP (n = 3) mice were randomly chosen and photographed at different time points throughout their development. Mice are age-matched at postnatal (p) day 4, p15, 4 weeks (wk), 7 wk, and 12 wk. (E) Survival curve of female WT (n = 4) and RdRP (n = 4) mice in the absence of infection. (F and G) WT (F, n = 2) and RdRP mice (G, n = 4) from (E) were photographed at 20 months of age.

In contrast, despite very high, constitutive ISG upregulation via activation of MDA5, RdRP mice have no evidence of autoimmunity or other systemic inflammatory conditions. Detailed analyses showed normal gross tissue morphology (Fig 4B) and histology (Fig 4A) with no detectable aberrant immune cell infiltration or inflammation. Kidneys had no glomerulonephritis (Fig 4C) as was found in MDA5 G821S mice [19]. In fact, RdRP mice are healthy and indistinguishable from WT controls throughout their development (Fig 4D) in all identifiable variables including habitus, weight gain, activity level, fertility, mating, and longevity (Fig 4E–4G). Effects of background strain are possible and could be empirically tested (e.g., MDA5 G821S in DBA/2J vs. RdRP-*Mda5* ^*-/-*^ in C57BL/6J/FVB mice here). Nevertheless, these results redefine the range of chronic innate immune system activation states that can be well-tolerated in mammals. That is, a highly augmented antiviral state based on constitutive MDA5-mediated ISG activation can persist without inflammatory disease or other deleterious sequelae.

### A catalytically-active RdRP is necessary and sufficient for induction of an antiviral phenotype in human cells

In cultured vertebrate cells, RdRP expression can induce antiviral cytokine production, but mechanisms operating at the RNA and protein levels have been proposed. For example, transfection of plasmids encoding a 430 nucleotide segment of the parainfluenza virus RdRP transcript activated MDA5-mediated IFNβ production, implicating a structural motif within the RNA transcript itself [15]. In contrast, expression of the Semliki Forest virus RdRP protein in transfected cells induced IFNβ via dsRNA generation by templating on host RNA [16].

**Fig 5:**
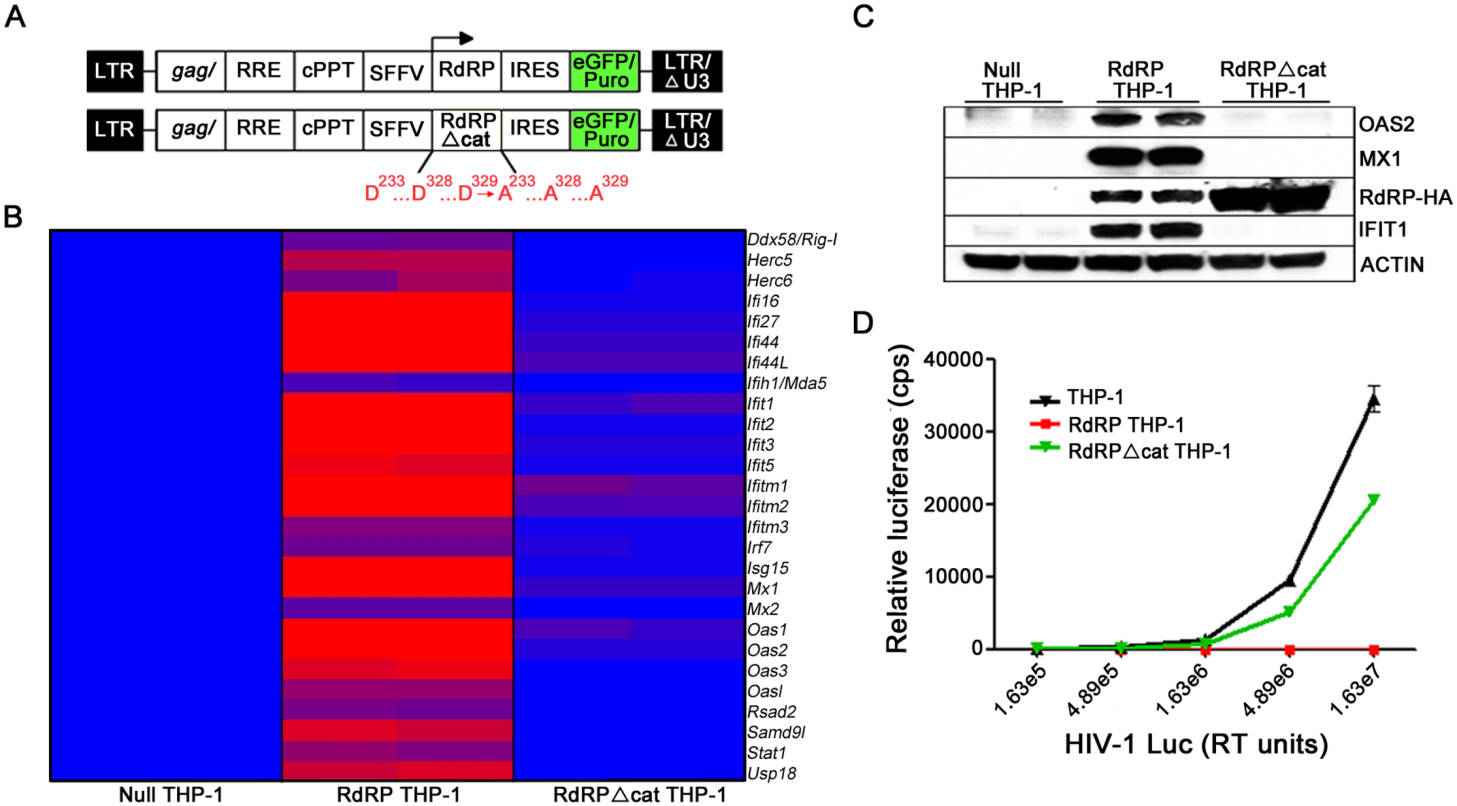
Human THP-1 cells expressing a catalytically-active RdRP display highly augmented antiviral defenses. (A) Stable RdRP THP-1 cell lines were generated using lentiviral vectors that co-express GFP-puro with RdRP, RdRPΔcat, or no RdRP (null). (B) Heat map of differentially expressed genes in THP-1 monocytes, presented as fold change (blue = 1-fold induction; purple = 8-fold upregulation; red = ≥15-fold upregulation). RdRP: subset of antiviral genes significantly upregulated >4-fold in RdRP THP-1 cells (n = 2) compared with null empty vector control THP-1 cells (n = 2). RdRPΔcat: THP-1 cells expressing the RdRPΔcat transgene (n = 2) compared with null empty vector control THP-1 cells (n = 2). For a complete list of upregulated genes see S4 & S7 Tables. (C) THP-1 cell lysates were run in duplicate and analyzed by immunoblotting using antibodies to OAS2, MX1, and IFIT1. Antibody to HA-tag was used to determine relative abundance of RdRP-HA protein in each cell line. Antibody to β-ACTIN served as control, data are representative of two independent experiments. (D) Infection by an HIV-1 reporter virus (HIV-1_luc_) in RdRP, RdRPΔcat, and parental THP-1 cell lines. Data are mean ± SEM of three technical replicates, and are representative of two independent experiments.

To determine the mechanism for innate immune activation and induction of constitutive antiviral signaling by the TMEV RdRP, we constructed mutant RdRP genes. First, we introduced pervasive, coding-neutral point mutations into the RdRP cDNA to maximally disrupt primary and secondary RNA structure (RdRPΔrna; S3A Fig). Another mutant, RdRPΔcat, lacks catalytic activity due to alanine substitutions of the three key catalytic center triad aspartate residues (D233, D328, and D329) [23,24,25], but is otherwise intact at the nucleotide and amino acid levels (Fig 5A). The WT RdRP, RdRPΔrna, and RdRPΔcat versions of the RdRP transgenes were transduced with lentiviral vectors into human THP-1 monocytes, with RdRP mRNA transcription controlled by the Spleen Focus Forming Virus (SFFV) promoter. In parallel, a control cell line transduced with a vector lacking any RdRP transgene (null THP-1) was generated. After selection in puromycin, whole-genome expression profiling was performed on total RNA extracted from the different THP-1 lines.

Significantly, THP-1 monocytes expressing the WT RdRP and RdRPΔrna highly upregulated (up to 560-fold) critical antiviral genes in a pattern that closely resembles that of RdRP mice (Fig 5B and S3B Fig, S4–S6 Tables), indicating that the reconfigured ISG network can be induced by the RdRP in human cells. Furthermore, a structural motif within the RdRP-encoding mRNA is not required for induction of an antiviral state (S3C and S3D Fig).

In contrast to the results with RdRPΔrna, THP-1 cells expressing the catalytically-inactive (RdRPΔcat) and control vector-transduced THP-1 cells did not highly upregulate antiviral effectors (Fig 5B and 5C, S7 Table). Since THP-1 cells are a key model for cellular innate immunity to HIV-1 [26], we challenged the THP-1 lines with this virus and observed that cells expressing the RdRP from either the WT or RdRPΔrna cDNAs were refractory to infection, while RdRPΔcat cells were not (Fig 5D and S3D Fig). Thus, an enzymatically-active RdRP, rather than structural motifs within the RdRP-encoding mRNA, is necessary for induction of an antiviral phenotype.

**Fig 6:**
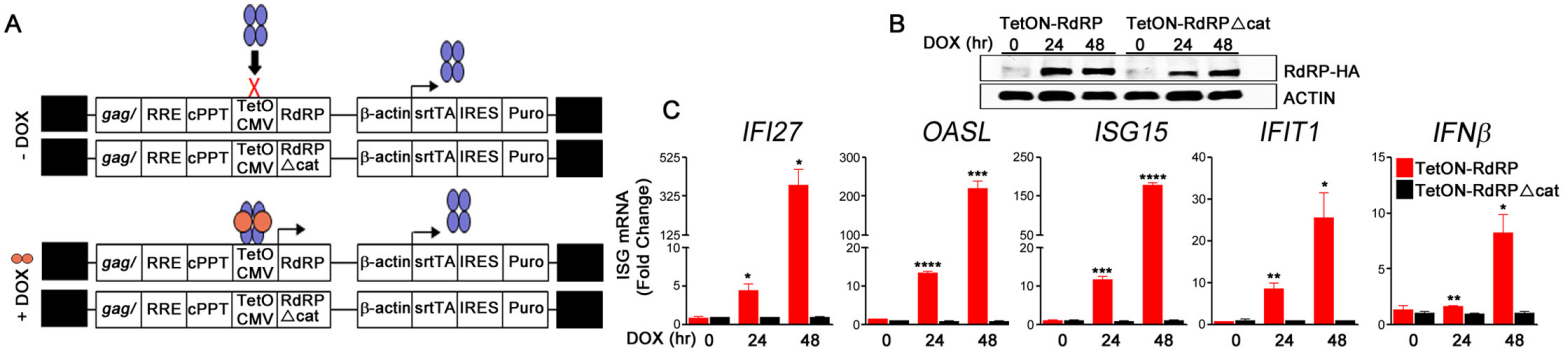
Rapid induction of antiviral ISG mRNAs following RdRP expression in human lung epithelial cells. (A) DOX-inducible RdRP A549 cell lines were generated using lentiviral vectors that inducibly co-express RdRP or RdRPΔcat and resistance to puromycin. Vector terminology: Tsin-TetON-RdRP-IRESpuro (TetON-RdRP) and Tsin-TetON-RdRPΔcat-IRESpuro (TetON-RdRPΔcat) lentiviral vectors. (B) TetON-A549 cell lysates were analyzed by immunoblotting to determine relative abundance of RdRP-HA protein (anti-HA-tag) following DOX treatment (hr). (C) mRNA levels of the prominent antiviral genes, *IFI27*, *OASL*, *ISG15*, *IFIT1*,and *IFNβ* in TetON-A549 cell lines prior to (0 hr) or after (24 and 48 hr) DOX treatment (data are mean ± SEM of three technical replicates, relative to *GAPDH* mRNA). * *P<0*.05; ** P<0.01; *** P<0.001; **** P<0.*0001*.

Additionally, when we expressed the RdRP inducibly in the human lung epithelial cell line A549, innate antiviral signaling pathways resulting in elevated ISG profiles were rapidly activated. For this group of experiments, we used lentiviral vectors that mediate doxycycline-inducible expression of the WT RdRP or the RdRPΔcat mutant (Fig 6A). Reverse transcriptase activity-normalized vectors were used to transduce A549 cells followed by selection in puromycin to produce stable lines. Doxycycline (DOX) was added to the media to induce RdRP or RdRPΔcat expression. Then, subsets of cells were harvested and assayed for ISG induction 24 and 48 hr after DOX treatment. Without DOX treatment (0 hr), there was no statistically-significant difference between basal levels of ISGs in the TetON-RdRP or TetON-RdRPΔcat A549 cell lines (Fig 6C). When the catalytically-active RdRP was expressed following DOX treatment (Fig 6B), ISGs were rapidly induced up to 380-fold (Fig 6C). However, elevated ISG mRNA profiles were not observed in the enzymatically-inactive TetON-RdRPΔcat cells following DOX treatment (Fig 6C). Collectively, these data confirm that an enzymatically-active RdRP is sufficient for induction of innate antiviral signaling in diverse RdRP-expressing human cell types.

### Identification of dsRNA in RdRP mouse tissue

PRR activation during viral infection can be triggered by long viral dsRNAs. Because the experiments described above showed that a catalytically active RdRP is necessary for ISG induction and antiviral protection, we hypothesized that the unsequestered RdRP-generated signal is dsRNA transcribed from cellular RNA templates. We used the anti-dsRNA K1 antibody to assay tissues of uninfected RdRP mice for the presence of endogenous dsRNA [27]. These experiments demonstrated that uninfected tissues of RdRP mice contain substantial endogenous dsRNA, which is located in the cytoplasmic compartment in a heterogeneous, focal pattern (S4 Fig).

## Discussion

During infection, positive-strand RNA viruses elaborately rearrange host-cell endoplasmic reticulum, Golgi, and lysosome membranes to create 50–500 nm vesicle clusters that house the viral synthetic apparatus [3]. These virus-induced rearrangements of host cell membranes were originally characterized based on their ability to increase efficiency and concentrate components of the viral replication process. Termed replication factories, these enclosures house viral proteases, viral genomes, replication intermediates, and RdRPs [3,28]. For picornaviruses, replication factory assembly is a complex, ordered process requiring viral translation, intracellular membrane rearrangement, vesicle budding and viral RNA synthesis. Studies of poliovirus have suggested that endoplasmic-reticulum derived vesicles coalesce to form rosette-shaped membrane structures with viral replicative intermediates sequestered within the complex [5,6,7]. To what extent these membranous structures prevent interaction of the picornavirus RdRP and its dsRNA products with cytosolic components (such as nucleic acid sensors) is unknown. However, plant experiments in the 1990s suggested that transgenic RdRP expression can activate innate immunity in tissues of a multicellular eukaryote [8,9,10,11,12,13]. Whether RdRP expression can similarly reconfigure innate immunity in vertebrate animals was previously not known.

Our present data show that transgenic RdRP expression in mice produces a quantitatively dramatic, sustained, effectively antiviral ISG network. The effect requires the MDA5-MAVS pathway, implying that dsRNA produced by the RdRP is the effective trigger. Indeed, we show the presence of endogenous dsRNA in neural tissue of uninfected RdRP mice (S4 Fig), and we hypothesize that this is due to nonspecific replicase activity similar to previously published reports of dsRNA generation following ectopic expression of viral RdRPs *in vitro*. For example, Nikonov and colleagues have shown that transient expression of the intact Semliki Forest virus RdRP protein activates IFNβ production in mammalian cell lines [16]. The authors determined that nonspecific replicase activity produced RNA PAMPs with the following characteristics: >200 nucleotides in length, non-polyadenylated, double-stranded, and containing a 5’-phosphate. The authors identified a putative RdRP-specific RNA clone, albeit within a mixture of PAMPs, that was complimentary to mouse antisense non-coding mitochondrial RNA 1, and they concluded that the Semliki Forest virus RdRP can induce IFNβ via dsRNA generation by templating on host RNA. Importantly, in this case sensing of dsRNA products was mediated through both RIG-I and MDA5 since knockdown of either sensor alone was not sufficient to completely abolish innate immune activation [16]. Similar to these results, we show that when the TMEV RdRP is expressed transgenically (in the complete absence of other viral proteins), the presence of endogenous dsRNA accompanies PRR-mediated innate immune activation. In contrast, however, our data demonstrate that knockout of MDA5 completely blocks TMEV RdRP-induced ISG activation (S2D Fig) which results in compromised murine survival following viral infection (Fig 3D). Therefore, we hypothesize the nonspecific TMEV replicase activity produces dsRNA by templating on host RNA, which in turn activates MDA5-MAVS-mediated ISG activation. However, a role for RIG-I in this system cannot be ruled out at present.

Taken together, transgenic RdRP-mediated innate immune activation supports an additional role for viral replication factories in addition to their function of concentrating and providing physical support for the viral synthetic apparatus. Since they isolate the RdRP and its viral dsRNA products from the cytosol, replication factories provide a barrier to detection by the host innate immune system. Thus, we propose that transgenic expression of the viral RdRP outside the viral context uncouples this enzyme from the elaborate membrane confinement program that is normally orchestrated by multiple viral proteins.

Our results further show that stable MAVS-dependent MDA5 signaling augments host antiviral defenses without triggering inflammatory disease. Thus, we propose that a powerfully augmented MDA5-mediated activation state can be a well-tolerated mammalian innate immune system configuration. This is a novel result, further investigation of which may provide clues to the pathogenesis of nucleic acid-based autoimmunity. The quintessential outcome of aberrant MDA5 activation has heretofore been documented in genetic association studies of autoimmune disorders and type I interferonopathies. Human SNPs in both MAVS [29] and MDA5 [30] carry increased risk of developing SLE. In a genome-wide association study, MDA5 was also identified as a susceptibility locus for autoimmune type 1 diabetes [31]. More recently, rare missense gain-of-function MDA5 mutations were shown to cause AGS, an inflammatory disorder primarily affecting the skin and nervous system. Affected individuals presented with a range of disease phenotypes in which elevated ISG expression due to enhanced interferon state was central to pathogenesis [18]. Similar clinical manifestations have also been reported in mice harboring a single missense mutation (G821S) in MDA5 resulting in an autoactivated form [19].

MDA5 G821S mice and RdRP mice share a common molecular feature: abnormal, constitutive MDA5-MAVS-mediated upregulation of ISGs. Of note however, MDA5 G821S mice showed elevated ISG levels in the presence of up to 300-fold elevated *Ifnβ* transcript and only partial dependence on the IFNαβR. In G821S mice, the authors proposed that the only partial dependence on IFNαβR suggests potential involvement of other inflammatory cytokines (IL-6, TNFα) that may amplify a signaling cascade leading to chronic effector T cell activation.

Conversely, in RdRP mice, elevated antiviral ISG levels exhibit a complete dependence on the IFNαβR (Fig 3B). We have previously detected 16-fold basal upregulation of *Ifnβ* mRNA in brain tissue of RdRP mice [17]. However, in the present study, we neither detected induction of *Ifnα* nor *Ifnβ* mRNAs in our microarray gene expression profiling of spinal cord tissue in which ISG mRNAs were highly elevated (S5A Fig and S2 Table). Furthermore, basal plasma type I IFN levels were not elevated (S5B Fig, 0 hr), and RdRP mice displayed attenuated type I IFN induction following administration of the dsRNA analogue, poly(I:C) (S5B Fig, 14 hr). In RdRP-expressing THP-1 monocytes, neither human *IFNα* nor *IFNβ* mRNAs were significantly upregulated in our gene expression profiling studies despite ISG mRNA induction of up to 560-fold (S4 Table). Inducible expression of the RdRP in A549 cells, however, resulted in an early wave (48 hr) of 8-fold *IFNβ* induction (Fig 6C).

In RdRP-transgenic mice and human cells, our data demonstrate that high, ubiquitous, potentially destructive chronic upregulation of type I IFN levels is not at play. Rather, we hypothesize that a dynamically regulated IFN environment may maintain the constitutively augmented RdRP-induced antiviral network, with IFNαβR-mediated signaling balanced by type I IFN negative regulators (such as *Usp18* [32], *Nlrc5* [33] and *Lgp2* [34], upregulated 93, 6, and 4-fold, respectively, in uninfected RdRP mice; S2 Table).

This work therefore revises an existing paradigm in which innate immune responses and ISG activation are temporally constrained—with failure to limit this response leading to chronic inflammatory disease. We contend that MDA5 signaling and associated ISG induction can be modulated to a set-point previously considered a flagrant emergency response or disease state, and that further study may reveal the mechanisms that permit RdRP mice to tolerate sustained MDA5-MAVS-mediated, highly augmented pathogen defenses without destructive autoimmunity or other chronic inflammatory sequelae.

Vaccines or effective drugs are not readily available for many emerging/re-emerging agents of zoonotic origin, and established countermeasures are likely to be equally lacking for infectious agents engineered with biowarfare intent. Conventional strategies to prevent and treat viral diseases generally focus on inducing antigen-specific adaptive immunity (vaccines) or targeting specific viral molecules (restriction factors, antiviral drugs). Such approaches must be tailored to each virus or to a limited group of closely related viruses and cannot broadly prevent infection or diseases caused by viruses from diverse families. The innate immune system is not antigen-specific but functions instead to sense a variety of viral motifs. Activation of the cell-intrinsic viral sensing pathway empowers broadly antiviral capabilities via ISG activation [35,36,37,38,39,40]. Consistent with these data, we have shown that augmenting host innate immune responses can stably (Fig 1D), safely (Fig 4), rapidly (Fig 6C), and broadly (EMCV, HIV-1, TMEV, vesicular stomatitis virus, pseudorabies virus) [17,20] protect against viral disease. Therapeutic stimulation of innate immune pathways in this way may adequately limit the replication and spread of novel virulent pathogens. Collectively, these data provide sufficient evidence to motivate future antiviral development endeavors that critically evaluate materials and methods for safely augmenting host innate immunity to achieve broad-spectrum viral attenuation.

## Materials and Methods

### Ethics statement

All animals were cared for according to local, state, and federal regulation in accordance with protocol approved by the Mayo Institutional Animal Care and Use Committee (A21313) and consistent with National Institutes of Health guidelines for the care and use of animals.

### Mice

Transgenic FVB RdRP^+/+^, VP1^+/+^, and VP2^+/+^ mice were generated as described previously [41]. *Rag1* ^-/-^ mice on a C57BL/6 background were originally obtained from the Jackson Laboratories and backcrossed >5 generations yielding *Rag1* ^-/-^ mice on a FVB background. *Tlr3* ^-/-^ (C57BL/6NJ; #009675), *Mavs* ^-/-^ (B6.129SF2/J; #008634), and *Mda5* ^-/-^ (*Ifih1* ^*-/-*^, C57BL/6J; #015812) mice were obtained from the Jackson Laboratories. *IfnαβR* ^*-/-*^ (*Ifnar1* ^-/-^, B6.129S2; #032045) were obtained from MMRRC. *IfnγR* ^*-/-*^(*Ifngr1* ^-/-^, G129) mice were maintained on their original 129Sv(ev) background [42]. Knockout mice (*Tlr3* ^-/-^, *Mavs* ^-/-^, *Mda5* ^-/-^, *IfnαβR* ^*-/-*^, or *IfnγR* ^*-/-*^) were bred to FVB RdRP^+/+^ mice, and the resulting heterozygotes were used to generate sublines. Mice used for RT-PCR and immunoblotting were directly compared with sibling controls (Fig 3 and S2 Fig). To obtain enough mice per genotype for survival curves (Fig 3), second generation homozygotes were intercrossed and bred to double homozygosity. Thus, in all sublines our data directly compare knockout RdRP^-/-^ mice derived from the same cross that generated the knockout RdRP^+/+^ mice. In genetic crosses, the RdRP transgene was inherited in ratios consistent with Mendelian inheritance. Age-matched adult mice (> 8 weeks) were used for experiments.

### RdRP THP-1 cell lines

The SFFV promoter, RdRP cDNAs, and a GFP-puromycin fusion protein selection marker were cloned into transfer constructs containing HIV LTRs, a small portion of gag, an RRE, and a cPPT. Vector was produced by three-part transfection as described previously [43]. Transfer constructs were transfected into HEK 293T cells along with packaging construct RΔ8.9 and envelope pseudotyping construct expressing VSV-G. Vectors were assayed for reverse transcriptase (RT) activity. THP-1 cells (ATCC TIB-202) were cultured in RPMI with glutamine + 10% FBS + 100U/ml penicillin + 10mM HEPES + 1X MEM-NEAA. RT-normalized amounts of vector were used to transduce THP-1 cells. Cells were washed at 16 hours post-transduction to remove excess. Transduced cells were selected in puromycin (3μg/ml) for approximately two weeks to produce stable cell lines expressing RdRP transgenes.

### TetON-RdRP A549 cells

Tsin IRESpuro [44] was digested with BamI and NheI and a 2.1 kb band containing IRESpuro was isolated. This was inserted into a BamHI and NheI digested Tsin-TetOn to position the IRESpuro downstream of the β-actin promoter to make Tsin-TetOn-IRESpuro. Tsin-TetOn-RdRP-IRESpuro and Tsin-TetOn-RdRPΔcat-IRESpuro were cloned by digesting Tsin-RdRP/RdRPΔcat-GFP-puromycin (described above) with PmeI and BamHI. The 1440 bp band was gel purified and treated with Klenow to create blunt ends, then ligated with Tsin-TetOn-IRESpuro that was blunt-end linearized with PmeI. Transfer constructs were transfected into HEK 293T cells along with packaging construct RΔ8.9 and envelope pseudotyping construct expressing VSV-G. Vectors were assayed for reverse transcriptase (RT) activity. A549 cells (ATCC CCL-185) were cultured in DMEM with glutamine + 10% FBS + 100U/ml penicillin + 10mM HEPES + 1X MEM-NEAA. RT-normalized amounts of vector were used to transduce A549 cells. Cells were washed at 16 hours post-transduction to remove excess. Transduced cells were selected in puromycin (1.5 μg/ml) for approximately two weeks to produce stable cell lines. DOX was added (1 μg/ml) to the media to induce RdRP or RdRPΔcat expression.

### Viruses

Mice were infected with 40 PFU of EMCV (ATCC#VR129B) by intraperitoneal injection [17]. RdRP THP-1 cell lines were plated at 2e5 cells/24 well and transduced with a VSV-G pseudotyped HIV-1 reporter virus, NL4-3 R- E- Δ426, which contains a 426 nt deletion in *env* and expresses luciferase (HIV-1_luc_). 72 hours post transduction, THP-1 cells were lysed in PBS-T (1% Tween 20 in PBS), and luciferase was assayed with SteadyGlo (Promega) in triplicate with a Packard TopCount NXT microplate luminescence counter.

### Gene chip microarrays

Microarray analyses of murine tissue investigated gene expression profiles of WT and transgenic RdRP mice during uninfected (baseline) conditions or following EMCV infection. For baseline gene expression studies, total RNA from spinal cords of age-matched, sex-matched adult WT FVB (n = 3) and RdRP FVB (n = 3) mice was isolated according to the manufacturer’s recommendations (Qiagen RNeasy kit). For expression studies following EMCV infection, age-matched WT or RdRP mice were sacrificed two days post infection and spinal cord RNA was isolated (n = 2 uninfected WT, n = 2 infected WT, n = 2 infected RdRP mice). RNA from each sample was used as a template to synthesize biotinylated cRNA which was then hybridized to the HT Mouse Genome 430 2.0 GeneChip Array (Affymetrix) at the Mayo Clinic Gene Expression Core facility (Rochester, MN). Raw data from cell intensity files (.cel) were analyzed using Partek Genomics Suite 6.6 (St. Louis, MO). Significant expression changes between treatment groups were identified using a fold change criterion of four-fold and a *P* value (two-tailed) of *< 0*.*05*.

Microarray analyses of THP-1 cells investigated gene expression profiles of cell lines stably-expressing different mutant forms of the RdRP transgene. Total RNA from each cell line (n = 2) was isolated according to the manufacturer’s recommendations (Qiagen RNeasy kit). RNA from each sample was used as a template to synthesize biotinylated cRNA which was then hybridized to the Human Genome U133 Plus 2.0 GeneChip Array (Affymetrix) at the Mayo Clinic Gene Expression Core facility (Rochester, MN). Raw data from cell intensity files (.cel) were analyzed using Partek Genomics Suite 6.6 (St. Louis, MO). Significant expression changes between cell lines were identified using a fold change criterion of four-fold and a *P* value (two-tailed) of < *0*.*05*.

### Microarray data deposition

The data discussed in this publication have been deposited at NCBI Gene Expression Omnibus (GEO, http://www.ncbi.nlm.nih.gov/geo) and are available through GEO SuperSeries accession number GSE62759.

### RT-PCR

Total RNA was extracted from homogenized tissues of age-matched adult mice according to the manufacturer’s recommendations (Qiagen RNeasy kit or Invitrogen Trizol Reagent). Total RNA was reverse transcribed and amplified in one step (Qiagen Quantifast SYBR Green RT PCR kit; Roche LightCycler 480). Primers were generated for the EMCV viral transcript (5’-GCCCGACCTCTGCTAAGATACTAACA—3’ and 5’- GCAAGTAAACTGGTTGAGCCACAAA -3’) mouse *Gapdh* (5’-AGATTGTTGCCATCAACGACCC -3’ and 5’- CTCGCTCCTGGAAGATGGTG -3’) the murine ISGs, *Ifit1* (5’-TTTGTTGTTGTTGTTGTTCGTT-3’ and 5’-GCAGGAATCAGTTGTGATCT -3’), *Oasl2* (5’- CAAACAAACAAACAAACCCTCTC -3’ and 5’- TCAAGGTGTCACTCTGCAT -3’), *Isg15* (5’- TGGTACAGAACTGCAGCGAG -3’ and 5’- CAGCCAGAACTGGTCTTCGT -3’), and *Rig-I* (5’- CCAAGAAATTACCAACTGGAGCTTGC -3’ and 5’- TGCTCATAGACAGGAATTTGGTTAGCGAA -3’), the human ISGs, *IFI27* (5’- ATCAGCAGTGACCAGTGTGG -3’ and 5’- GGCCACAACTCCTCCAATCA -3’), *OASL* (5’-GGATCTTCTCCCACACTCACATCT -3’ and 5’- CACCATCAGGATTCTTCACGAA -3’), *ISG15* (5’- GTGGACAAATGCGACGAACC -3’ and 5’- ATTTCCGGCCCTTGATCCTG -3’), *IFIT1* (5’- AGCTTACACCATTGGCTGCT -3’ and 5’- CCATTTGTACTCATGGTTGCTGT -3’), human *IFNβ* (5’- AGTAGGCGACACTGTTCGTG -3’ and 5’- AGCCTCCCATTCAATTGCCA -3’), and human *GAPDH* (5’- TGGACCTGACCTGCCGT -3’ and 5’- TGGAGGAGTGGGTGTCGC -3’). Samples were run at least in duplicate, and mean crossing point for each transcript was determined and normalized to *Gapdh* (deltaCt). DeltaCt values were then used to calculate mRNA fold-change relative to *Gapdh* using the 2^-[delta][delta]Ct method[45]. *P* values were calculated using the Student’s two-tailed unpaired *t* test (Graphpad Prism). *P* values *< 0*.*05* were considered significant.

### Survival curves

Age-matched, sex-matched adult mice were used to assess survival following EMCV infection. During experiments, health status of the mice was monitored and moribund mice were euthanized in accordance with the Mayo Institutional Animal Care and Use Committee and consistent with National Institutes of Health guidelines for the care and use of animals. *P* values were calculated using the log rank Mantel-Cox test (Graphpad Prism). *P* values *< 0*.*05* were considered significant.

### Western blotting

Cerebrum, spinal cord and cerebellum tissue (30–50 mg) were harvested from age-matched adult mice. Tissues were homogenized in lysis buffer at a final concentration of 150 μg tissue/μl buffer [50 mM Tris-HCl, 5 mM EDTA, 150 mM NaCl, 1% Triton X100, SDS 0.05%, pH 4.4, 1mM Na_3_VO_4_, and 10 mM NaF, supplemented with protease inhibitor cocktail (Roche cOmplete ULTRA tablets)]. Tissue lysates were centrifuged at 19600 x g for 30 minutes to remove insoluble material. 25 μl of protein sample was diluted in reducing XT SDS sample buffer (Bio-Rad). Samples were separated by 4–20% gradient SDS-Page, transferred to polyvinylidene difluoride membrane, and blocked for 1 hour at room temperature (5% dry milk in phosphate buffered saline supplemented with 0.05% Tween 20; PBS-T). The membrane was then incubated at 4°C overnight in diluted primary antibody (5% BSA in PBS-T). Rabbit polyclonal antibodies (Cell Signaling) for RIG-I (#3743; 1:1000 dilution), ISG15 (#2743; 1:1000 dilution), and β-ACTIN (#4967; 1:1000 dilution) were detected with goat anti-rabbit secondary antibodies conjugated to horseradish peroxidase (1:5000, Millipore) and developed with Western Lightning Plus-ECL Chemiluminescence Substrate (Perkin Elmer).

Human THP-1 cells and human A549 cells were lysed as described above. Total protein was measured by BCA assay (Thermo Scientific Pierce) and protein-normalized samples were diluted in reducing XT SDS sample buffer (Bio-Rad). Samples were separated by 10% gradient SDS-Page. Rabbit polyclonal antibodies for OAS2 (Abcam ab90045; 1:1000 dilution), IFIT1 (Cell Signaling #12082; 1:1000), MX1 (Abcam ab95926; 1:1000) and β-ACTIN (Cell Signaling #4967; 1:1000) were detected with goat anti-rabbit secondary antibodies conjugated to horseradish peroxidase (Millipore; 1:5000) and developed with Western Lightning Plus-ECL Chemiluminescence Substrate (Perkin Elmer). The anti-HA tag rat monoclonal antibody (Roche 1867423; 1:2000) used to detect HA-tagged RdRP protein and was probed with goat anti-rat secondary antibodies (1:5000, Millipore) conjugated to horseradish peroxidase.

### ELISA

Serum samples from adult age-matched, sex-matched mice were taken before (n = 3 per genotype) or 14 hours after (n = 5 per genotype) a 100 ug intraperitoneal injection of polyinosine-polycytidylic acid (high molecular weight polyI:C; InvivoGen). IFNα and IFNβ production were measured using commercial ELISA kits according to manufacturer’s protocol (Mouse IFNα Platinum ELISA, eBioscience; VeriKine Mouse IFNβ ELISA kit, PBL interferon source). Serum samples were run in duplicate and mean OD levels were used to calculate interferon concentration. Interferon levels below the assay detection limit (IFNα 31.3 pg/ml, IFNβ 15.6 pg/ml) were recorded as not detected. Significant differences between WT and RdRP interferon concentrations were assessed using the Student’s two-tailed unpaired *t* test (Graphpad Prism). *P* values *< 0*.*05* were considered significant.

### Histological analysis

Tissues from age-matched, sex-matched uninfected adult mice were fixed in 4% paraformaldehyde, paraffin embedded, cut into sections, and stained with hematoxylin-eosin. Slides were photographed using an Olympus DP73 camera attached to an Olympus AX70 microscope.

### RNA in situ hybridization

Brain tissue from uninfected age-matched, sex-matched mice was fixed in 4% paraformaldehyde. Paraffin embedded tissues were hybridized with labeled probes to visualize expression of antiviral ISGs (*Ifit1* and *Ifi27l2a*) using the commercial Quantigene ViewRNA ISH Tissue 2-Plex Assay kit according to manufacturer’s protocol (Affymetrix). Probes to visualize *Gapdh* were included as control. Slides were photographed using an Olympus DP73 camera attached to an Olympus AX70 microscope or Olympus LEICA DM 25000 confocal microscope equipped with a 40x immersion objective and analyzed with LAS AF 6000 acquisition software (Leica Microsystems). All of the images compared were obtained using the same instrument settings.

### dsRNA immunohistochemistry

Brain tissues from uninfected age-matched, sex-matched mice were flash-frozen in liquid nitrogen. Tissue was cryosectioned at 10μm (Leica Cryostat CM1860). Sections were kept on ice and fixed for 10 minutes using Carnoy’s solution (25% v/v glacial acetic acid, 75% v/v absolute ethanol) and then rinsed for 10 minutes in distilled water. Tissues were incubated (5 min) with peroxidase block (Dako), and stained with the K1 anti-dsRNA antibody (English and Scientific Consulting, Hungary; 1:200 dilution in PBS, 30 minute incubation) that had been biotinylated using the Dako ARK peroxidase kit for mouse primary antibodies (Dako, K3955). Biotinylated-K1 antibody was detected using the streptavidin-peroxidase (30 minute incubation) and DAB+substrate-chromogen (3 minute incubation) reagents (Dako). Slides were photographed using an Olympus DP73 camera attached to an Olympus AX70 microscope. All of the images compared were obtained using the same instrument settings.

## Supporting Information

S1 Fig RdRP mice inherently express antiviral effectors. (A) Venn diagram summary of murine microarray analyses. Each treatment group (WT+EMCV, uninfected RdRP, or RdRP+EMCV) was compared to the respective uninfected WT control group included in that experiment. Orange: gene upregulated >4-fold in the WT+EMCV treatment group only. Red: genes upregulated >4-fold in the uninfected RdRP treatment group only. Purple: genes upregulated >4-fold in the RdRP+EMCV treatment group only. Blue: genes upregulated >4-fold in all three treatment groups. Gray: gene upregulated >4-fold in the WT+EMCV and uninfected RdRP treatment groups. Green: genes upregulated >4-fold in the uninfected RdRP and RdRP+EMCV treatment groups. For a complete list of upregulated genes see S1–S3 Tables. (B) RT-PCR analysis of the prominent antiviral genes, *Oasl2*, *Isg15*, *Ifit1*, and *Rig-I* in lung, kidney, heart, and liver tissues of uninfected FVB mice (n = 3 mice per genotype, relative to *Gapdh* mRNA, mean ± SEM). (C) (Left) Hematoxylin-eosin staining. (Right) In situ RNA hybridization for expression of antiviral ISGs (red; *Ifit1* and *Ifi27l2a* co-labeling) in different brain regions of uninfected WT and RdRP mice (scale bar 50 μm). All of the images compared were obtained using the same instrument settings. (D) RT-PCR analysis of ISGs in cerebrum, spinal cord, and cerebellum tissues of uninfected FVB mice (n = 5 per genotype, relative to *Gapdh* mRNA, mean ± SEM). (E) Cerebrum, spinal cord, and cerebellum tissue homogenates (n = 2 uninfected mice per genotype, 1 per lane) were analyzed by immunoblotting using antibodies for RIG-I and ISG15. Antibody for β-ACTIN served as control. (F) RT-PCR analysis of viral titers in murine tissues two days post infection with EMCV (n = 9 mice per genotype, relative to *Gapdh* mRNA, mean ± SEM). (G) Survival curves (Kaplan-Meier plot) following EMCV infection (n = 8 mice per genotype). Data are representative of two independent experiments. * P<0.05; ** P<0.01; *** P<0.001; **** P<0.*0001*.
(TIF)

S2 Fig RdRP-induced ISG inductions are mediated in a MDA5-MAVS, IFNαβR-dependent manner. (A-E) Knockout mice were bred to RdRP mice and the antiviral ISGs, *Oasl2*, *Isg15*, *Ifit1*, and *Rig-I*, were analyzed by RT-PCR in CNS tissues of uninfected mice. Mice used were directly compared with sibling controls (n = 5 per genotype, relative to *Gapdh* mRNA, mean ± SEM). *P<0.*05*, **P<0.*01*, ***P<0.*001*, ****P<0.*0001*.
(TIF)

S3 Fig THP-1 monocytes expressing the RdRP from mRNA with pervasively altered nucleotide sequence display highly augmented antiviral defenses. (A) RdRP cDNA was synthesized with coding-neutral point mutations that maximally disrupt primary and secondary RNA structure (RdRPΔrna). Human codon usage was employed. The WT viral RdRP sequence is shown in the top line. (B) Stable RdRP THP-1 cell lines were generated using lentiviral vectors that co-express GFP-puro with RdRPΔrna or no RdRP (null). Lines were selected in puromycin. Heat map of differentially expressed genes in THP-1 monocytes, presented as fold change (blue = 1-fold induction; purple = 5.5-fold upregulation; red = ≥10-fold upregulation). RdRPΔrna: Subset of antiviral genes significantly upregulated >4-fold in RdRPΔrna THP-1 cells (n = 2) compared with null empty vector control THP-1 cells (n = 2). For a complete list of upregulated genes see S5 Table. (C) THP-1 cell lysates were run in duplicate and analyzed by immunoblotting using antibodies to OAS2, MX1, and IFIT1. Antibody to β-ACTIN served as control, data are representative of two independent experiments. (D) Infectivity of firefly luciferase-expressing HIV-1 virus (HIV-1_luc_) in RdRPΔrna and parental THP-1 cell lines, data are mean ± SEM of three technical replicates.
(TIF)

S4 Fig RdRP mouse tissues express endogenous dsRNA. Expression of endogenous dsRNA (biotinylated K1 antibody, peroxidase stain) in brains of uninfected WT and RdRP mice. Slides were photographed using an Olympus DP73 camera attached to an Olympus AX70 microscope (left panels 50 μm, right 20 μm).
(TIF)

S5 Fig IFNαβR-dependent antiviral signaling despite an attenuated type I IFN response in RdRP mice. (A) Results from microarray analysis. Gene expression profile of type I IFNs (*Ifna1*, Refseq ID NM_010502; *Ifna2*, NM_010503; *Ifna4*, NM_010504; *Ifna5*, NM_010505; *Ifna9* NM_010507; *Ifna11*, NM_008333; *Ifnab* NM_008336; *Ifnb1*, NM_010510) in spinal cords of uninfected RdRP mice (n = 3) compared with uninfected WT mice (n = 3), data are representative of two independent experiments. (B) ELISA analysis of type I IFN levels in murine serum samples before (0 hours, n = 3 per genotype) or 14 hours after (n = 5 per genotype) a single intraperitoneal injection of the synthetic dsRNA analog, poly(I:C). Interferon levels below the assay detection limit (IFNα 31.3 pg/ml, IFNβ 15.6 pg/ml) are indicated as not detected (nd).
(TIF)

S1 Table List of genes upregulated in virally-infected wildtype mice. Differential gene expression in spinal cords from wildtype (WT) mice infected with EMCV (n = 2) compared to uninfected WT mice (n = 2). Gene chip was analyzed as described in methods. Only genes with a fold change in expression >4.0 and a p-value of <0.05 are shown. There were no genes significantly downregulated in EMCV-infected mice. References to gene expression made in the body of the paper represent the most upregulated probeset related to that gene.
(PDF)

S2 Table List of genes upregulated in uninfected RdRP mice. Differential gene expression in spinal cords from uninfected RdRP mice (n = 3) compared to uninfected WT mice (n = 3). Gene chip was analyzed as described in methods. Only genes with a fold change in expression >4.0 and a p-value of <0.05 are shown. There were no genes significantly downregulated. References to gene expression made in the body of the paper represent the most upregulated probeset related to that gene.
(PDF)

S3 Table List of genes differentially expressed in virally-infected RdRP mice. Differential gene expression in spinal cords from RdRP mice infected with EMCV (n = 2 females) compared to uninfected WT mice (n = 2 males). Genes listed in italics represent sex-linked genes. Gene chip was analyzed as described in methods. Only genes with a fold change in expression >4.0 or <-4.0 and a p-value of <0.05 are shown. References to gene expression made in the body of the paper represent the most upregulated probeset related to that gene.
(PDF)

S4 Table List of genes differentially expressed in RdRP THP-1 cells. Differential gene expression in THP-1 cells expressing the RdRP transgene (n = 2) compared to THP-1 empty vector control cells (n = 2). Gene chip was analyzed as described in methods. Only genes with a fold change in expression >4.0 or <-4.0 and a p-value of <0.05 are shown. References to gene expression made in the body of the paper represent the most upregulated probeset related to that gene.
(PDF)

S5 Table List of genes differentially expressed in codon-optimized RdRP THP-1 cells. Differential gene expression in THP-1 cells expressing a codon-optimized RdRP, RdRPΔrna, transgene (n = 2) compared to THP-1 empty vector control cells (n = 2). Gene chip was analyzed as described in methods. Only genes with a fold change in expression >4.0 or <-4.0 and a p-value of <0.05 are shown. References to gene expression made in the body of the paper represent the most upregulated probeset related to that gene.
(PDF)

S6 Table RdRP expression triggers an innate antiviral response in uninfected hosts. Murine genes upregulated (>4-fold) in tissues of uninfected RdRP mice and human genes upregulated (>4-fold) in RdRP-expressing THP-1 monocytes encode proteins with prominent roles in broad-spectrum viral attenuation.
(PDF)

S7 Table List of genes differentially expressed in RdRPΔcat THP-1 cells. Differential gene expression in THP-1 cells expressing the catalytic mutant, RdRPΔcat, transgene (n = 2) compared to THP-1 empty vector control cells (n = 2). Gene chip was analyzed as described in methods. Only genes with a fold change in expression >4.0 or <-4.0 and a p-value of <0.05 are shown. References to gene expression made in the body of the paper represent the most upregulated probeset related to that gene.
(PDF)

S1 References References cited in Supporting Information files. (DOCX)
