# Supplementary figures and images for: Antiviral Protection via RdRP-Mediated Stable Activation of Innate Immunity
Source: PLoS Pathog. 2015 Dec 3;11(12):e1005311. doi: 10.1371/journal.ppat.1005311 (PMC4669089; doi:10.1371/journal.ppat.1005311)

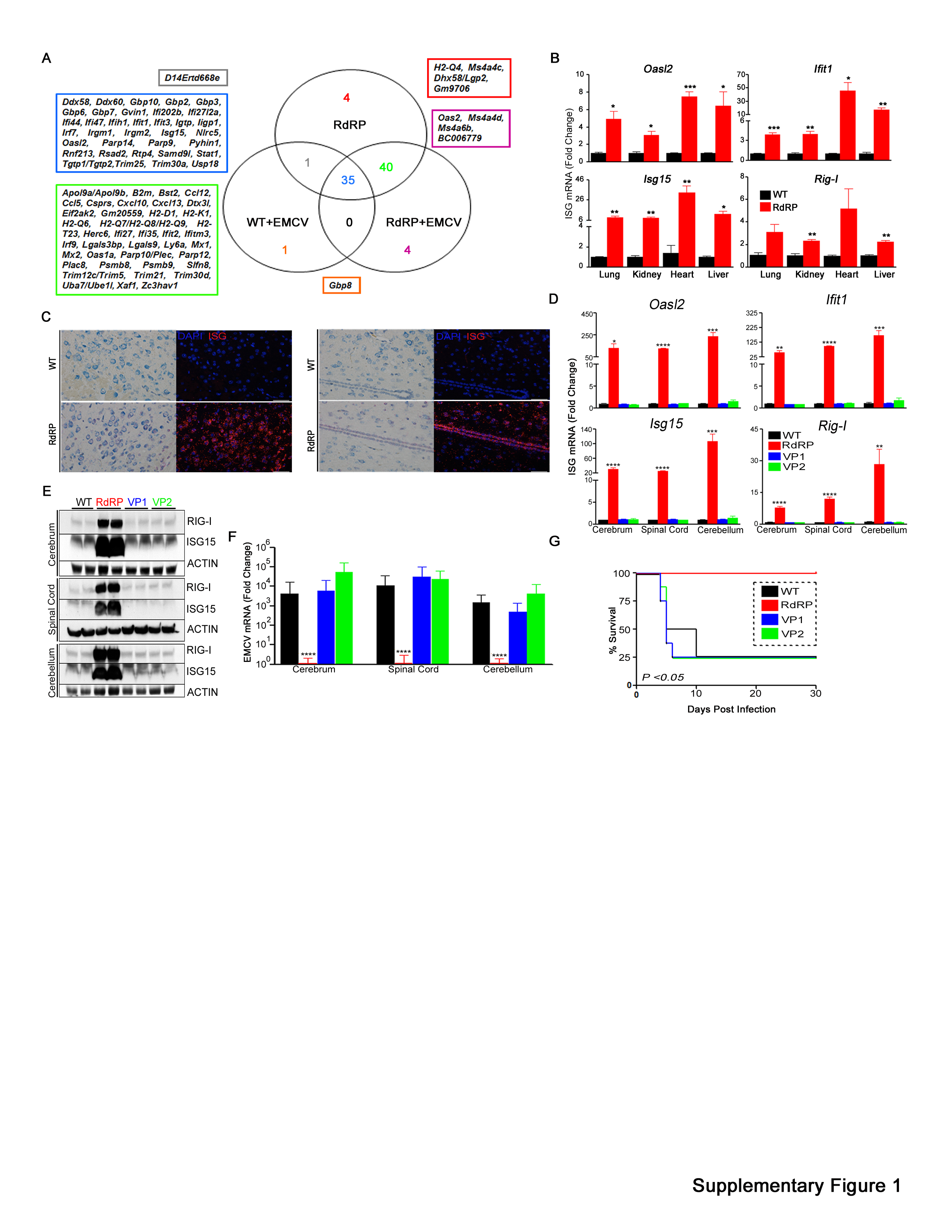

Supplement: S1 Fig — (A) Venn diagram summary of murine microarray analyses. Each treatment group (WT+EMCV, uninfected RdRP, or RdRP+EMCV) was compared to the respective uninfected WT control group included in that experiment. Orange: gene upregulated >4-fold in the WT+EMCV treatment group only. Red: genes upregulated >4-fold in the uninfected RdRP treatment group only. Purple: genes upregulated >4-fold in the RdRP+EMCV treatment group only. Blue: genes upregulated >4-fold in all three treatment groups. Gray: gene upregulated >4-fold in the WT+EMCV and uninfected RdRP treatment groups. Green: genes upregulated >4-fold in the uninfected RdRP and RdRP+EMCV treatment groups. For a complete list of upregulated genes see S1–S3 Tables. (B) RT-PCR analysis of the prominent antiviral genes, Oasl2, Isg15, Ifit1, and Rig-I in lung, kidney, heart, and liver tissues of uninfected FVB mice (n = 3 mice per genotype, relative to Gapdh mRNA, mean ± SEM). (C) (Left) Hematoxylin-eosin staining. (Right) In situ RNA hybridization for expression of antiviral ISGs (red; Ifit1 and Ifi27l2a co-labeling) in different brain regions of uninfected WT and RdRP mice (scale bar 50 μm). All of the images compared were obtained using the same instrument settings. (D) RT-PCR analysis of ISGs in cerebrum, spinal cord, and cerebellum tissues of uninfected FVB mice (n = 5 per genotype, relative to Gapdh mRNA, mean ± SEM). (E) Cerebrum, spinal cord, and cerebellum tissue homogenates (n = 2 uninfected mice per genotype, 1 per lane) were analyzed by immunoblotting using antibodies for RIG-I and ISG15. Antibody for β-ACTIN served as control. (F) RT-PCR analysis of viral titers in murine tissues two days post infection with EMCV (n = 9 mice per genotype, relative to Gapdh mRNA, mean ± SEM). (G) Survival curves (Kaplan-Meier plot) following EMCV infection (n = 8 mice per genotype). Data are representative of two independent experiments. * P<0.05; ** P<0.01; *** P<0.001; **** P<0.0001. (TIF) [file ppat.1005311.s001.tif]

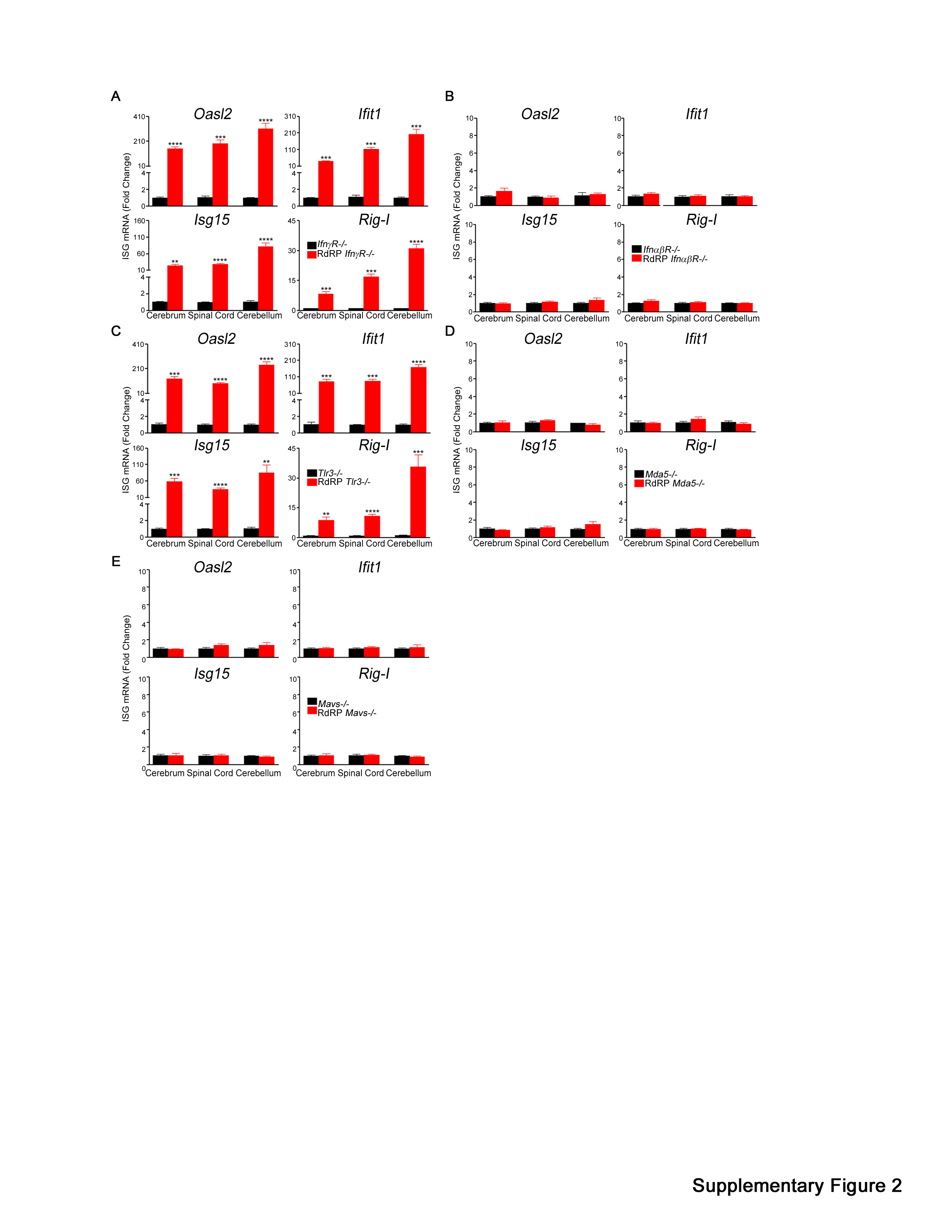

Supplement: S2 Fig — (A-E) Knockout mice were bred to RdRP mice and the antiviral ISGs, Oasl2, Isg15, Ifit1, and Rig-I, were analyzed by RT-PCR in CNS tissues of uninfected mice. Mice used were directly compared with sibling controls (n = 5 per genotype, relative to Gapdh mRNA, mean ± SEM). *P<0.05, **P<0.01, ***P<0.001, ****P<0.0001. (TIF) [file ppat.1005311.s002.tif]

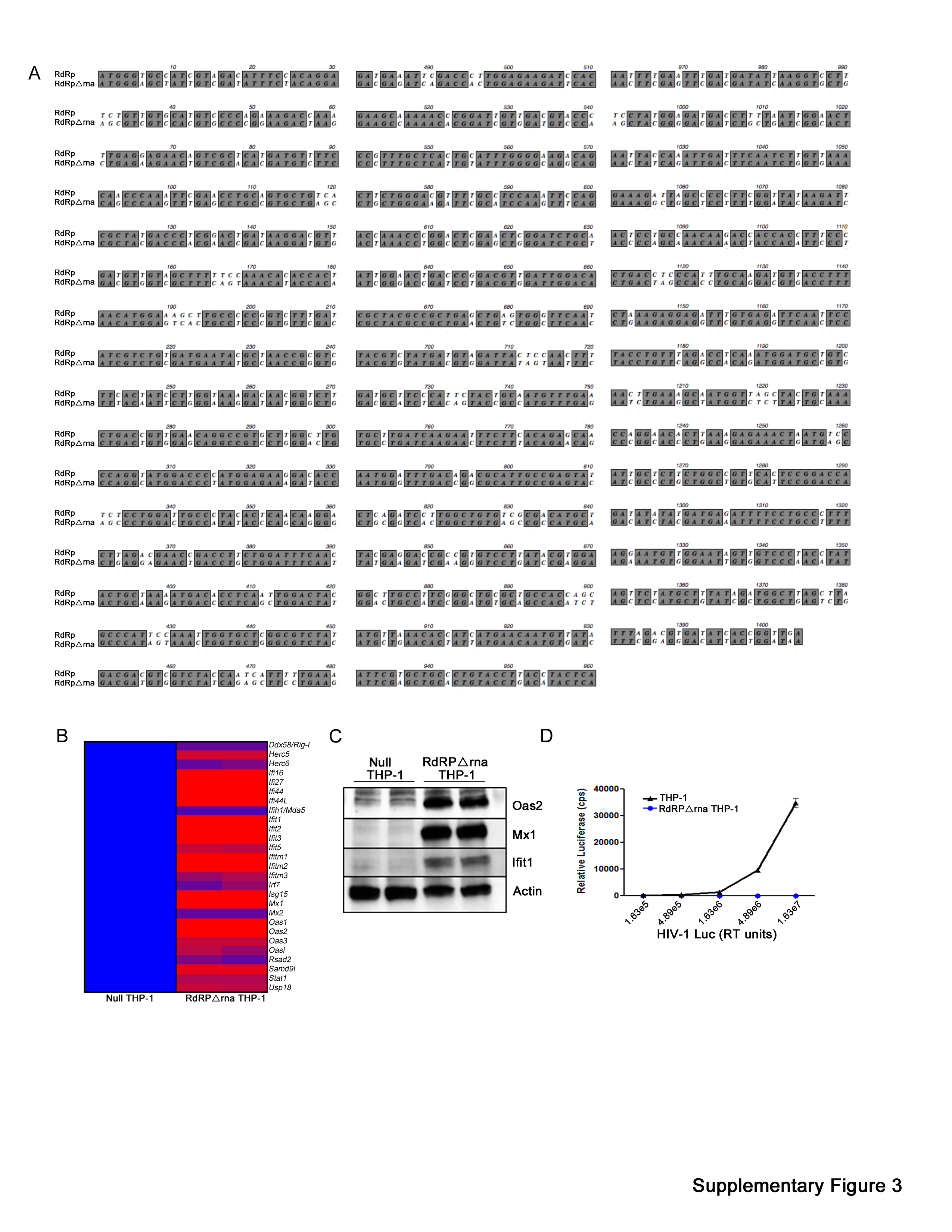

Supplement: S3 Fig — (A) RdRP cDNA was synthesized with coding-neutral point mutations that maximally disrupt primary and secondary RNA structure (RdRPΔrna). Human codon usage was employed. The WT viral RdRP sequence is shown in the top line. (B) Stable RdRP THP-1 cell lines were generated using lentiviral vectors that co-express GFP-puro with RdRPΔrna or no RdRP (null). Lines were selected in puromycin. Heat map of differentially expressed genes in THP-1 monocytes, presented as fold change (blue = 1-fold induction; purple = 5.5-fold upregulation; red = ≥10-fold upregulation). RdRPΔrna: Subset of antiviral genes significantly upregulated >4-fold in RdRPΔrna THP-1 cells (n = 2) compared with null empty vector control THP-1 cells (n = 2). For a complete list of upregulated genes see S5 Table. (C) THP-1 cell lysates were run in duplicate and analyzed by immunoblotting using antibodies to OAS2, MX1, and IFIT1. Antibody to β-ACTIN served as control, data are representative of two independent experiments. (D) Infectivity of firefly luciferase-expressing HIV-1 virus (HIV-1luc) in RdRPΔrna and parental THP-1 cell lines, data are mean ± SEM of three technical replicates. (TIF) [file ppat.1005311.s003.tif]

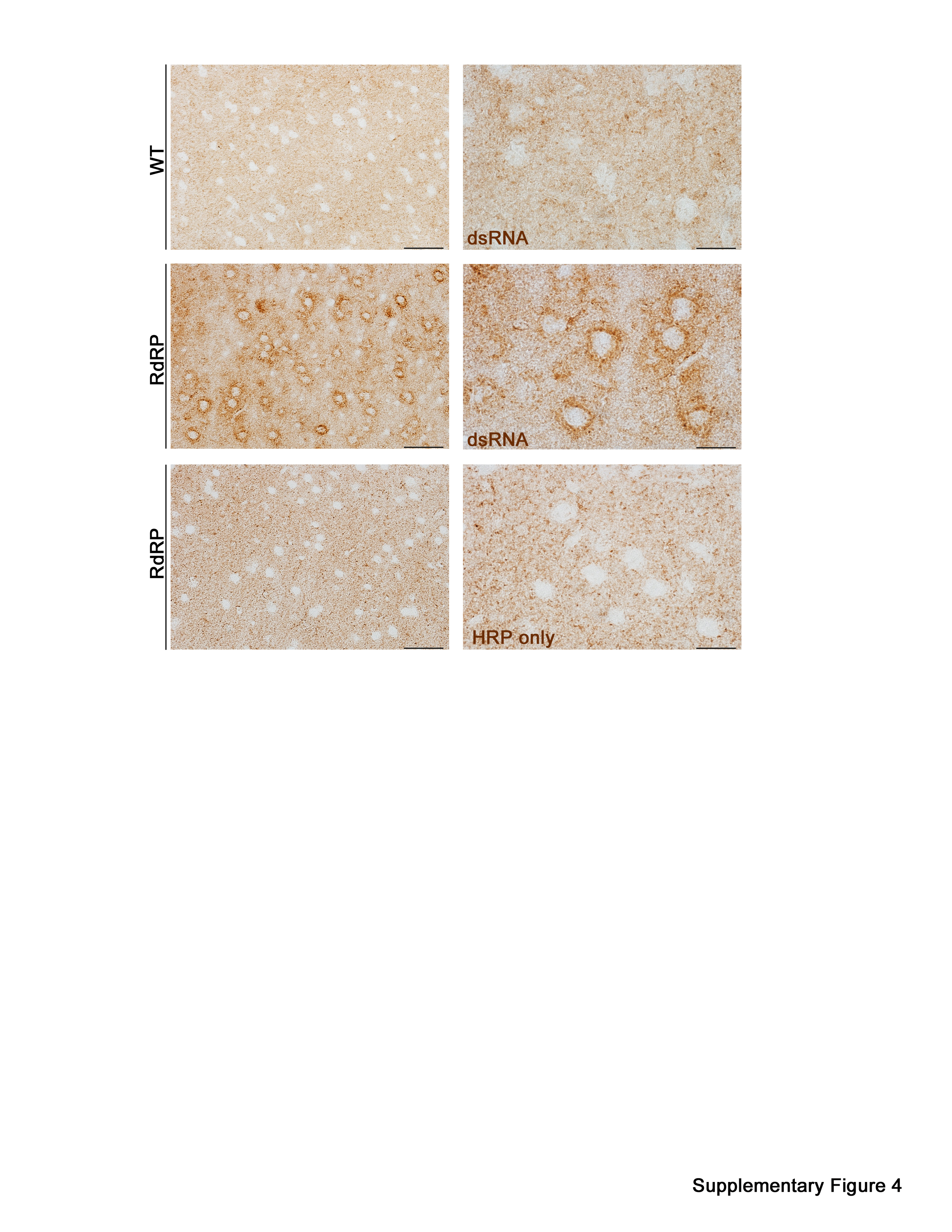

Supplement: S4 Fig — Expression of endogenous dsRNA (biotinylated K1 antibody, peroxidase stain) in brains of uninfected WT and RdRP mice. Slides were photographed using an Olympus DP73 camera attached to an Olympus AX70 microscope (left panels 50 μm, right 20 μm). (TIF) [file ppat.1005311.s004.tif]

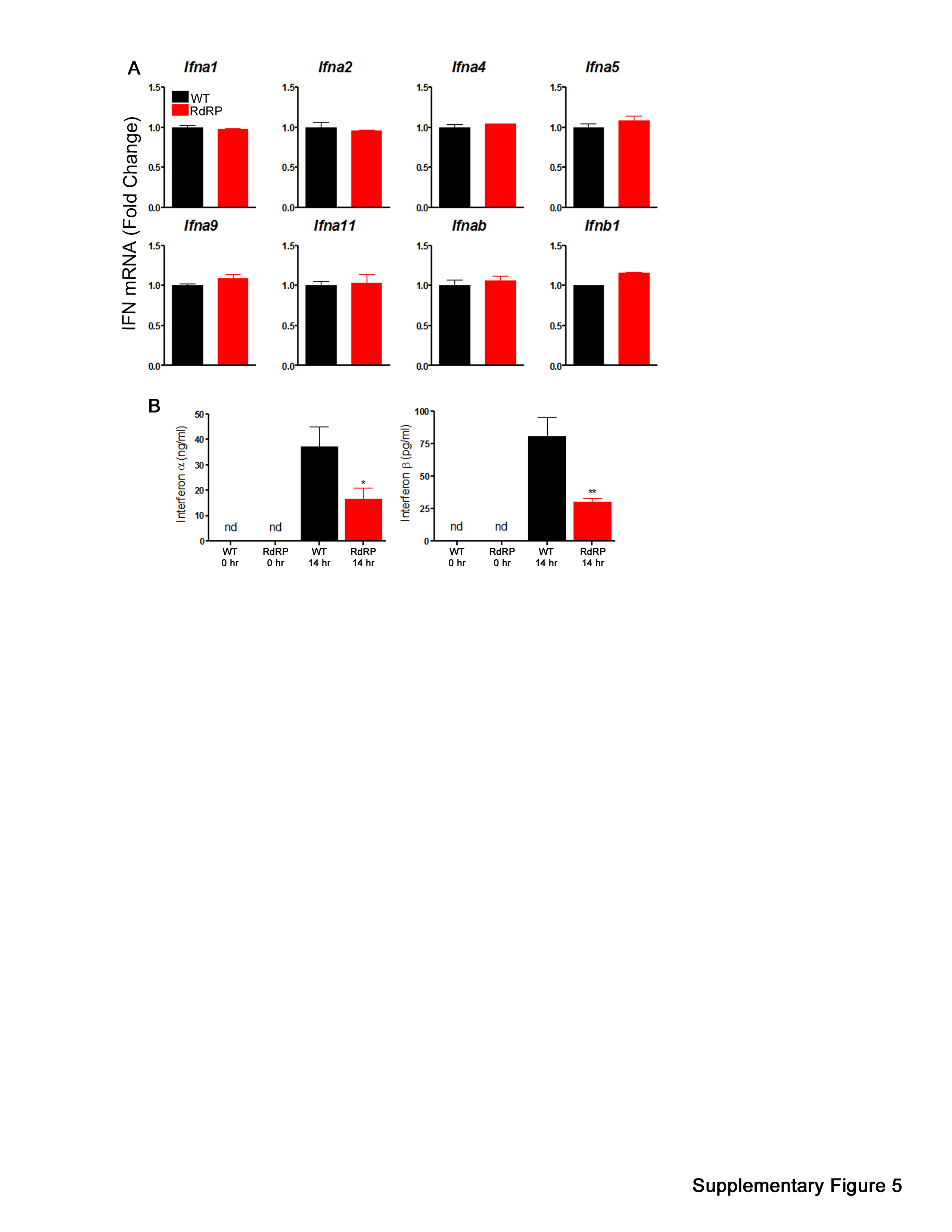

Supplement: S5 Fig — (A) Results from microarray analysis. Gene expression profile of type I IFNs (Ifna1, Refseq ID NM_010502; Ifna2, NM_010503; Ifna4, NM_010504; Ifna5, NM_010505; Ifna9 NM_010507; Ifna11, NM_008333; Ifnab NM_008336; Ifnb1, NM_010510) in spinal cords of uninfected RdRP mice (n = 3) compared with uninfected WT mice (n = 3), data are representative of two independent experiments. (B) ELISA analysis of type I IFN levels in murine serum samples before (0 hours, n = 3 per genotype) or 14 hours after (n = 5 per genotype) a single intraperitoneal injection of the synthetic dsRNA analog, poly(I:C). Interferon levels below the assay detection limit (IFNα 31.3 pg/ml, IFNβ 15.6 pg/ml) are indicated as not detected (nd). (TIF) [file ppat.1005311.s005.tif]
