# Supplementary material for: Antiviral Protection via RdRP-Mediated Stable Activation of Innate Immunity
Source: PLoS Pathog. 2015 Dec 3;11(12):e1005311. doi: 10.1371/journal.ppat.1005311 (PMC4669089; doi:10.1371/journal.ppat.1005311)
Supplement: S1 Table — Differential gene expression in spinal cords from wildtype (WT) mice infected with EMCV (n = 2) compared to uninfected WT mice (n = 2). Gene chip was analyzed as described in methods. Only genes with a fold change in expression >4.0 and a p-value of <0.05 are shown. There were no genes significantly downregulated in EMCV-infected mice. References to gene expression made in the body of the paper represent the most upregulated probeset related to that gene. (PDF) [file ppat.1005311.s006.pdf]

S1 Table. List of genes upregulated in virally-infected wildtype mice. Differential gene expression in spinal cords from wildtype (WT) mice infected with EMCV (n=2) compared to uninfected WT mice (n=2). Gene chip was analyzed as described in methods. Only genes with a fold change in expression >4.0 and a p-value of <0.05 are shown. There were no genes significantly downregulated in EMCV-infected mice. References to gene expression made in the body of the paper represent the most upregulated probeset related to that gene.

Probeset ID	Gene Symbol	Gene Title	RefSeq ID	Fold Change	P-value
1418191_at	Usp18	ubiquitin specific peptidase 18	NM_011909	26.53	6.84E-03
1450783_at	Ifit1	interferon-induced protein with tetratricopeptide repeats 1	NM_008331	23.85	4.06E-03
1449009_at	Tgtp1 /// Tgtp2	T cell specific GTPase 1, 2	NM_011579 /// NM_001145164	22.40	1.13E-02
1449025_at	Ifit3	interferon-induced protein with tetratricopeptide repeats 3	NM_010501	18.87	2.35E-03
1457666_s_at	Ifi202b	interferon activated gene 202B	NM_008327	17.94	8.63E-03
1423555_a_at	Ifi44	interferon-induced protein 44	NM_133871	15.45	7.45E-03
1419043_a_at	Iigp1	interferon inducible GTPase 1	NM_001146275	15.20	1.16E-02
1419042_at	Iigp1	interferon inducible GTPase 1	NM_001146275	13.65	9.63E-03
1453196_a_at	Oasl2	2'-5' oligoadenylate synthetase-like 2	NM_011854	12.43	6.63E-03
1418580_at	Rtp4	receptor transporter protein 4	NM_023386	11.03	2.98E-03
1431591_s_at	Isg15	ISG15 ubiquitin-like modifier	NM_015783	10.72	6.08E-03
1451860_a_at	Trim30a	tripartite motif-containing 30A	NM_009099	10.37	5.14E-03
1426278_at	Ifi27l2a	interferon, alpha-inducible protein 27 like 2A	NM_029803	9.42	4.27E-03
1418392_a_at	Gbp3	guanylate binding protein 3	NM_018734	8.64	3.18E-03
1417793_at	Irgm2	immunity-related GTPase family M member 2	NM_019440	7.43	1.21E-02
1439831_at	---	---	---	7.24	1.19E-02
1434380_at	Gbp7	guanylate binding protein 7	NM_001083312	6.93	8.91E-03
1421009_at	Rsad2	radical S-adenosyl methionine domain containing 2	NM_021384	6.81	3.99E-03
1435331_at	Pyhin1	pyrin and HIN domain family, member 1	NM_175026	6.31	2.10E-02
1417141_at	Igtp	interferon gamma induced GTPase	NM_018738	6.22	1.64E-02
1429184_at	Gvin1	GTPase, very large interferon inducible 1	NM_001039160	6.21	1.19E-02
1418240_at	Gbp2	guanylate binding protein 2	NM_010260	6.05	2.58E-02
1436058_at	Rsad2	radical S-adenosyl methionine domain containing 2	NM_021384	5.92	9.10E-03
1438676_at	Gbp6	guanylate binding protein 6	NM_194336	5.75	2.43E-02
1425156_at	Gbp7	guanylate binding protein 7	NM_001083312	5.56	8.35E-03
1437176_at	Nlrc5	NLR family, CARD domain containing 5	NM_001033207	5.27	1.69E-02
1435906_x_at	Gbp2	guanylate binding protein 2	NM_010260	5.14	2.88E-02
1447927_at	Gbp10	guanylate-binding protein 10	NM_001039646	5.07	2.02E-02
1451777_at	Ddx60	DEAD (Asp-Glu-Ala-Asp) box polypeptide 60	NM_001081215	4.88	2.59E-02
1438868_at	D14Ertd668e	DNA segment, Chr 14, ERATO Doi 668, expressed	NM_001164323	4.87	4.77E-03
1460603_at	Samd9l	sterile alpha motif domain containing 9-like	NM_010156	4.75	4.93E-03
1451564_at	Parp14	poly (ADP-ribose) polymerase family, member 14	NM_001039530	4.69	2.89E-02
1426276_at	Ifih1	interferon induced with helicase C domain 1	NM_001164477	4.61	1.85E-02
1417244_a_at	Irf7	interferon regulatory factor 7	NM_001252600	4.52	1.99E-02
1421551_s_at	Ifi202b	interferon activated gene 202B	NM_008327	4.28	3.21E-02
1418776_at	Gbp8	guanylate-binding protein 8	NM_029509	4.27	3.60E-02
1436562_at	Ddx58	DEAD (Asp-Glu-Ala-Asp) box polypeptide 58	NM_172689	4.26	3.24E-03
1455500_at	Rnf213	ring finger protein 213	NM_001040005	4.25	1.31E-02
1417292_at	Ifi47	interferon gamma inducible protein 47	NM_008330	4.19	1.47E-02
1425974_a_at	Trim25	tripartite motif-containing 25	NM_009546	4.18	3.82E-03
1416897_at	Parp9	poly (ADP-ribose) polymerase family, member 9	NM_030253	4.10	9.52E-03
1450034_at	Stat1	signal transducer and activator of transcription 1	NM_001205313	4.10	4.14E-03
1418825_at	Irgm1	immunity-related GTPase family M member 1	NM_008326	4.09	1.07E-02
1450033_a_at	Stat1	signal transducer and activator of transcription 1	NM_001205313	4.04	5.59E-03
