# Supplementary material for: Antiviral Protection via RdRP-Mediated Stable Activation of Innate Immunity
Source: PLoS Pathog. 2015 Dec 3;11(12):e1005311. doi: 10.1371/journal.ppat.1005311 (PMC4669089; doi:10.1371/journal.ppat.1005311)
Supplement: S3 Table — Differential gene expression in spinal cords from RdRP mice infected with EMCV (n = 2 females) compared to uninfected WT mice (n = 2 males). Genes listed in italics represent sex-linked genes. Gene chip was analyzed as described in methods. Only genes with a fold change in expression >4.0 or <-4.0 and a p-value of <0.05 are shown. References to gene expression made in the body of the paper represent the most upregulated probeset related to that gene. (PDF) [file ppat.1005311.s008.pdf]

S3 Table. List of genes differentially expressed in virally-infected RdRP mice. Differential gene expression in spinal cords from RdRP mice infected with EMCV (n=2 females) compared to uninfected WT mice (n=2 males). Genes listed in italics represent sex-linked genes. Gene chip was analyzed as described in methods. Only genes with a fold change in expression >4.0 or <-4.0 and a p-value of <0.05 are shown. References to gene expression made in the body of the paper represent the most upregulated probeset related to that gene.

Probeset ID	Gene Symbol	Gene Title	RefSeq ID	Fold Change	P-value
1418191_at	Usp18	ubiquitin specific peptidase 18	NM_011909	124.48	1.86E-03
1426278_at	Ifi27l2a	interferon, alpha-inducible protein 27 like 2A	NM_029803	106.79	3.43E-04
1453196_a_at	Oasl2	2'-5' oligoadenylate synthetase-like 2	NM_011854	103.12	1.56E-04
1436936_s_at	*Xist*	*inactive X specific transcripts*	*NR_001463*	*91.58*	*1.80E-05*
1450783_at	Ifit1	interferon-induced protein with tetratricopeptide repeats 1	NM_008331	80.50	8.84E-04
1423555_a_at	Ifi44	interferon-induced protein 44	NM_133871	78.90	1.27E-03
1427262_at	*Xist*	*inactive X specific transcripts*	*NR_001463*	*54.37*	*4.73E-03*
1439831_at	---	---	---	41.81	4.62E-04
1431591_s_at	Isg15	ISG15 ubiquitin-like modifier	NM_015783	40.74	1.72E-03
1427263_at	*Xist*	*inactive X specific transcripts*	*NR_001463*	*36.79*	*5.34E-04*
1419043_a_at	Iigp1	interferon inducible GTPase 1	NM_001146275	36.17	2.06E-04
1419042_at	Iigp1	interferon inducible GTPase 1	NM_001146275	29.81	1.09E-04
1418580_at	Rtp4	receptor transporter protein 4	NM_023386	29.62	7.19E-04
1449025_at	Ifit3	interferon-induced protein with tetratricopeptide repeats 3	NM_010501	28.65	3.50E-04
1418392_a_at	Gbp3	guanylate binding protein 3	NM_018734	28.47	2.21E-04
1457666_s_at	Ifi202b	interferon activated gene 202B	NM_008327	27.45	2.04E-03
1424518_at	Apol9a /// Apol9b	apolipoprotein L 9a, 9b	NM_001162883 /// NM_001168660	25.67	1.65E-03
1451777_at	Ddx60	DEAD (Asp-Glu-Ala-Asp) box polypeptide 60	NM_001081215	24.50	8.96E-03
1438676_at	Gbp6	guanylate binding protein 6	NM_194336	23.22	5.28E-04
1451860_a_at	Trim30a	tripartite motif-containing 30A	NM_009099	22.88	1.03E-03
1449009_at	Tgtp1 /// Tgtp2	T cell specific GTPase 1, 2	NM_001145164 /// NM_011579	19.36	3.84E-03
1447927_at	Gbp10	guanylate-binding protein 10	NM_001039646	17.43	4.32E-05
1424775_at	Oas1a	2'-5' oligoadenylate synthetase 1A	NM_145211	16.84	3.29E-03
1421009_at	Rsad2	radical S-adenosyl methionine domain containing 2	NM_021384	15.74	4.17E-04
1460603_at	Samd9l	sterile alpha motif domain containing 9-like	NM_010156	13.82	2.56E-04
1436058_at	Rsad2	radical S-adenosyl methionine domain containing 2	NM_021384	13.50	1.93E-04
1431008_at	H2-Q6	histocompatibility 2, Q region locus 6	NM_207648	11.47	7.91E-04
1417793_at	Irgm2	immunity-related GTPase family M member 2	NM_019440	11.42	1.66E-04
1451335_at	Plac8	placenta-specific 8	NM_139198	11.39	7.59E-04
1434380_at	Gbp7	guanylate binding protein 7	NM_001083312	11.25	7.92E-04
1450034_at	Stat1	signal transducer and activator of transcription 1	NM_001205313	10.53	2.65E-04
1450033_a_at	Stat1	signal transducer and activator of transcription 1	NM_001205313	9.41	1.20E-03
1418930_at	Cxcl10	chemokine (C-X-C motif) ligand 10	NM_021274	9.40	6.00E-04
1443698_at	Xaf1	XIAP associated factor 1	NM_001037713	9.11	7.38E-04
1451655_at	Slfn8	schlafen 8	NM_001167743	8.99	4.84E-02
1438037_at	Herc6	hect domain and RLD 6	NM_025992	8.97	3.06E-04
1418825_at	Irgm1	immunity-related GTPase family M member 1	NM_008326	8.94	8.00E-04
1435331_at	Pyhin1	pyrin and HIN domain family, member 1	NM_175026	8.92	1.58E-02
1448380_at	Lgals3bp	lectin, galactoside-binding, soluble, 3 binding protein	NM_011150	8.81	2.60E-03
1440481_at	Stat1	signal transducer and activator of transcription 1	NM_001205313	8.81	8.93E-03
1421551_s_at	Ifi202b	interferon activated gene 202B	NM_008327	8.77	4.65E-03
1417141_at	Igtp	interferon gamma induced GTPase	NM_018738	8.59	4.13E-04
1435792_at	Csprs	component of Sp100-rs	NM_033616	8.53	1.50E-02
1425156_at	Gbp7	guanylate binding protein 7	NM_001083312	8.52	1.71E-03
1436562_at	Ddx58 /// Rig-I	DEAD (Asp-Glu-Ala-Asp) box polypeptide 58	NM_172689	8.34	1.45E-03
1426276_at	Ifih1 /// Mda5	interferon induced with helicase C domain 1	NM_001164477	8.30	1.21E-04
1420915_at	Stat1	signal transducer and activator of transcription 1	NM_001205313	8.12	8.97E-04
1456890_at	Ddx58 /// Rig-I	DEAD (Asp-Glu-Ala-Asp) box polypeptide 58	NM_172689	7.65	1.16E-03

1445897_s_at	Ifi35	interferon-induced protein 35	NM_027320	7.52	3.41E-04
1449556_at	H2-T23	histocompatibility 2, T region locus 23	NM_010398	7.43	9.21E-04
1417244_a_at	Irf7	interferon regulatory factor 7	NM_001252600	7.38	5.15E-05
1417185_at	Ly6a	lymphocyte antigen 6 complex, locus A	NM_010738	7.31	6.54E-04
1452178_at	Parp10 /// Plec	poly (ADP-ribose) polymerase family, member 10 /// plectin	NM_001163540 /// NM_001163575	7.25	7.17E-03
1451564_at	Parp14	poly (ADP-ribose) polymerase family, member 14	NM_001039530	7.22	8.87E-03
1449143_at	Rtp4	receptor transporter protein 4	NM_023386	7.19	1.59E-02
1424921_at	Bst2	bone marrow stromal cell antigen 2	NM_198095	7.04	2.37E-03
1421322_a_at	Irf9	interferon regulatory factor 9	NM_001159417	6.94	1.20E-04
1436183_at	Zc3hav1	zinc finger CCCH type, antiviral 1	NM_028421	6.89	7.18E-04
1417961_a_at	Trim30a	tripartite motif-containing 30A	NM_009099	6.72	1.18E-02
1436172_at	Gm20559	predicted gene, 20559	XR_104969	6.70	5.62E-03
1435665_at	Trim30d	tripartite motif-containing 30D	NM_001167828	6.66	6.86E-04
1437176_at	Nlrc5	NLR family, CARD domain containing 5	NM_001033207	6.53	1.55E-03
1435208_at	Dtx3l	deltex 3-like (Drosophila)	NM_001013371	6.44	4.08E-04
1455500_at	Rnf213	ring finger protein 213	NM_001040005	6.36	7.70E-06
1416897_at	Parp9	poly (ADP-ribose) polymerase family, member 9	NM_030253	6.31	2.38E-04
1443858_at	Trim12c /// Trim5	tripartite motif-containing 12C /// tripartite motif-containing 5	NM_001146007	6.29	6.79E-05
1425336_x_at	H2-K1	histocompatibility 2, K1, K region	NM_001001892	6.13	2.50E-04
1424948_x_at	H2-D1 /// H2-K1	histocompatibility 2, D region locus 1 /// K1, K region	NM_001001892 /// NM_010380	6.04	3.80E-04
1452956_a_at	Ifi27	interferon, alpha-inducible protein 27 like 1	NM_026790	5.99	1.97E-05
1426324_at	H2-D1	histocompatibility 2, D region locus 1	NM_010380	5.98	2.27E-04
1454757_s_at	Ifi27	interferon, alpha-inducible protein 27 like 1	NM_026790	5.93	2.80E-04
1423754_at	Ifitm3	interferon induced transmembrane protein 3	NM_025378	5.90	5.74E-04
1419879_s_at	Trim25	tripartite motif-containing 25	NM_009546	5.89	2.07E-03
1425974_a_at	Trim25	tripartite motif-containing 25	NM_009546	5.76	4.88E-04
1438027_at	---	---	---	5.65	1.63E-04
1421217_a_at	Lgals9	lectin, galactose binding, soluble 9	NM_001159301	5.64	3.31E-03
1449289_a_at	B2m	beta-2 microglobulin	NM_009735	5.64	1.27E-03
1440866_at	Eif2ak2	eukaryotic translation initiation factor 2-alpha kinase 2	NM_011163	5.53	3.38E-02
1426774_at	Parp12	poly (ADP-ribose) polymerase family, member 12	NM_172893	5.52	3.84E-03
1419282_at	Ccl12	chemokine (C-C motif) ligand 12	NM_011331	5.49	5.89E-03
1425065_at	Oas2	2'-5' oligoadenylate synthetase 2	NM_145227	5.46	1.36E-03
1418293_at	Ifit2	interferon-induced protein with tetratricopeptide repeats 2	NM_008332	5.37	1.22E-03
1429184_at	Gvin1	GTPase, very large interferon inducible 1	NM_001039160	5.33	1.20E-03
1427746_x_at	H2-K1	histocompatibility 2, K1, K region	NM_001001892	5.26	2.58E-04
1452428_a_at	B2m	beta-2 microglobulin	NM_009735	5.10	4.26E-04
1417851_at	Cxcl13	chemokine (C-X-C motif) ligand 13	NM_018866	5.09	3.26E-02
1418536_at	H2-Q7 /// Q8 /// Q9	histocompatibility 2, Q region locus 7, 8, 9	NM_001198560 /// NM_023124 /// NM_001201460	5.05	1.38E-03
1451683_x_at	H2-D1	histocompatibility 2, D region locus 1	NM_010380	5.01	5.24E-04
1439825_at	Dtx3l	deltex 3-like (Drosophila)	NM_001013371	5.01	1.10E-03
1418240_at	Gbp2	guanylate binding protein 2	NM_010260	5.01	2.80E-04
1448940_at	Trim21	tripartite motif-containing 21	NM_001082552	4.95	9.20E-04
1451905_a_at	Mx1	myxovirus (influenza virus) resistance 1	NM_010846	4.84	1.23E-02
1418990_at	Ms4a4d	membrane-spanning 4-domains, subfamily A, member 4D	NM_025658	4.71	2.64E-03
1424617_at	Ifi35	interferon-induced protein 35	NM_027320	4.71	4.06E-04
1417292_at	Ifi47	interferon gamma inducible protein 47	NM_008330	4.69	4.69E-03
1442201_at	---	---	---	4.67	1.44E-02
1435906_x_at	Gbp2	guanylate binding protein 2	NM_010260	4.67	2.40E-03
1422962_a_at	Psmb8	proteasome (prosome, macropain) subunit, beta type 8	NM_010724	4.59	2.30E-03
1450696_at	Psmb9	proteasome (prosome, macropain) subunit, beta type 9	NM_013585	4.44	2.23E-03

1418826_at	Ms4a6b	membrane-spanning 4-domains, subfamily A, member 6B	NM_027209	4.44	3.82E-03
1459151_x_at	Ifi35	interferon-induced protein 35	NM_027320	4.33	6.91E-04
1451931_x_at	H2-K1	Histocompatibility 2, K1, K region	NM_001001892	4.26	1.57E-04
1426971_at	Uba7 /// Ube1l	ubiquitin-like modifier activating enzyme 7	NM_023738	4.13	2.00E-03
1435454_a_at	BC006779	cDNA sequence BC006779	NM_183162	4.13	1.21E-03
1419676_at	Mx2	myxovirus (influenza virus) resistance 2	NM_013606	4.12	7.53E-03
1418126_at	Ccl5	chemokine (C-C motif) ligand 5	NM_013653	4.02	7.05E-04
**Genes downregulated in RdRP+EMCV mice**					
1424903_at	*Kdm5d*	*lysine (K)-specific demethylase 5D*	*NM_011419*	*-11.79*	*1.93E-02*
1426598_at	*Uty*	*ubiquitously transcribed tetratricopeptide repeat, Y chrom*	*NM_009484*	*-15.86*	*5.14E-04*
1417210_at	*Eif2s3y*	*eukaryotic translation initiation factor 2, Y-linked*	*NM_012011*	*-27.89*	*8.56E-04*
1452077_at	*Ddx3y*	*DEAD (Asp-Glu-Ala-Asp) box polypeptide 3, Y-linked*	*NM_012008*	*-32.39*	*2.43E-03*
1426439_at	*Ddx3y*	*DEAD (Asp-Glu-Ala-Asp) box polypeptide 3, Y-linked*	*NM_012008*	*-33.03*	*6.48E-04*
1426438_at	*Ddx3y*	*DEAD (Asp-Glu-Ala-Asp) box polypeptide 3, Y-linked*	*NM_012008*	*-115.81*	*2.12E-03*
