# Supplementary material for: Antiviral Protection via RdRP-Mediated Stable Activation of Innate Immunity
Source: PLoS Pathog. 2015 Dec 3;11(12):e1005311. doi: 10.1371/journal.ppat.1005311 (PMC4669089; doi:10.1371/journal.ppat.1005311)
Supplement: S4 Table — Differential gene expression in THP-1 cells expressing the RdRP transgene (n = 2) compared to THP-1 empty vector control cells (n = 2). Gene chip was analyzed as described in methods. Only genes with a fold change in expression >4.0 or <-4.0 and a p-value of <0.05 are shown. References to gene expression made in the body of the paper represent the most upregulated probeset related to that gene. (PDF) [file ppat.1005311.s009.pdf]

S4 Table. List of genes differentially expressed in RdRP THP-1 cells. Differential gene expression in THP-1 cells expressing the RdRP transgene (n=2) compared to THP-1 empty vector control cells (n=2). Gene chip was analyzed as described in methods. Only genes with a fold change in expression >4.0 or <-4.0 and a p-value of <0.05 are shown. References to gene expression made in the body of the paper represent the most upregulated probeset related to that gene.

Probeset ID	Gene Symbol	Gene Title	RefSeq ID	Fold Change	P-value
202411_at	IFI27	interferon, alpha-inducible protein 27	NM_001130080	565.32	2.15E-03
204439_at	IFI44L	interferon-induced protein 44-like	NM_006820	283.91	2.82E-04
214453_s_at	IFI44	interferon-induced protein 44	NM_006417	101.07	2.51E-04
213293_s_at	TRIM22	tripartite motif containing 22	NM_001199573	99.31	9.66E-04
202086_at	MX1	myxovirus (influenza virus) resistance 1	NM_001144925	76.07	3.06E-05
203153_at	IFIT1	interferon-induced protein with tetratricopeptide repeats 1	NM_001548	69.46	1.76E-04
227609_at	EPSTI1	epithelial stromal interaction 1 (breast)	NM_001002264	40.50	5.94E-05
201601_x_at	IFITM2	interferon induced transmembrane protein 2	NM_006435	39.01	1.69E-04
204972_at	OAS2	2'-5'-oligoadenylate synthetase 2, 69/71kDa	NM_001032731	36.00	1.54E-04
202686_s_at	AXL	AXL receptor tyrosine kinase	NM_001699	31.52	3.04E-04
214022_s_at	IFITM1	interferon induced transmembrane protein 1	NM_003641	30.54	1.59E-05
235276_at	EPSTI1	epithelial stromal interaction 1 (breast)	NM_001002264	28.87	9.89E-04
204415_at	IFI6	interferon, alpha-inducible protein 6	NM_002038	27.61	1.23E-04
202869_at	OAS1	2'-5'-oligoadenylate synthetase 1, 40/46kDa	NM_001032409	27.27	2.93E-03
205552_s_at	OAS1	2'-5'-oligoadenylate synthetase 1, 40/46kDa	NM_001032409	23.15	7.11E-04
229450_at	IFIT3	interferon-induced protein with tetratricopeptide repeats 3	NM_001031683	22.77	1.27E-04
219519_s_at	SIGLEC1	sialic acid binding Ig-like lectin 1, sialoadhesin	NM_023068	20.05	9.12E-03
226757_at	IFIT2	interferon-induced protein with tetratricopeptide repeats 2	NM_001547	18.96	8.70E-05
208965_s_at	IFI16	interferon, gamma-inducible protein 16	NM_001206567	17.58	7.14E-04
205483_s_at	ISG15	ISG15 ubiquitin-like modifier	NM_005101	16.88	5.39E-04
200923_at	LGALS3BP	lectin, galactoside-binding, soluble, 3 binding protein	NM_005567	14.93	1.16E-05
211122_s_at	CXCL11	chemokine (C-X-C motif) ligand 11	NM_005409	14.15	1.58E-03
218400_at	OAS3	2'-5'-oligoadenylate synthetase 3, 100kDa	NM_006187	12.73	1.91E-04
203595_s_at	IFIT5	interferon-induced protein with tetratricopeptide repeats 5	NM_012420	12.60	3.97E-04
204747_at	IFIT3	interferon-induced protein with tetratricopeptide repeats 3	NM_001031683	12.22	2.99E-04
204533_at	CXCL10	chemokine (C-X-C motif) ligand 10	NM_001565	11.64	4.07E-03
235643_at	SAMD9L	sterile alpha motif domain containing 9-like	NM_152703	11.59	1.10E-04
219211_at	USP18	ubiquitin specific peptidase 18	NM_017414	11.59	4.46E-03
228607_at	OAS2	2'-5'-oligoadenylate synthetase 2, 69/71kDa	NM_001032731	11.54	3.71E-05
214059_at	IFI44	Interferon-induced protein 44	NM_006417	11.08	4.69E-03
223220_s_at	PARP9	poly (ADP-ribose) polymerase family, member 9	NM_001146102	10.49	3.61E-04
219863_at	HERC5	HECT and RLD domain containing E3 ubiquitin protein ligase 5	NM_016323	10.05	1.51E-04
230036_at	SAMD9L	sterile alpha motif domain containing 9-like	NM_152703	9.41	2.92E-03
226603_at	SAMD9L	sterile alpha motif domain containing 9-like	NM_152703	9.09	2.50E-04
206133_at	XAF1	XIAP associated factor 1	NM_017523	9.03	2.68E-03
220059_at	STAP1	signal transducing adaptor family member 1	NM_012108	8.92	1.98E-03
216565_x_at	---	---	---	8.79	2.64E-03
205660_at	OASL	2'-5'-oligoadenylate synthetase-like	NM_001261825	8.46	2.40E-05
219352_at	HERC6	HECT and RLD domain containing E3 ubiquitin protein ligase 6	NM_001013000	8.45	1.89E-03
209969_s_at	STAT1	signal transducer and activator of transcription 1, 91kDa	NM_007315	8.20	5.93E-04
212203_x_at	IFITM3	interferon induced transmembrane protein 3	NM_021034	8.04	6.99E-06
242625_at	RSAD2	radical S-adenosyl methionine domain containing 2	NM_080657	7.69	3.51E-05
208436_s_at	IRF7	interferon regulatory factor 7	NM_001572	7.30	1.25E-03
218943_s_at	DDX58	DEAD (Asp-Glu-Ala-Asp) box polypeptide 58	NM_014314	7.18	1.09E-03
214255_at	ATP10A	ATPase, class V, type 10A	NM_024490	7.16	1.12E-03
226702_at	CMPK2	cytidine monophosphate (UMP-CMP) kinase 2, mitochondrial	NM_001256477	7.15	1.91E-03
223980_s_at	SP110	SP110 nuclear body protein	NM_001185015	7.05	8.64E-04
208966_x_at	IFI16	interferon, gamma-inducible protein 16	NM_001206567	7.03	2.44E-04
221969_at	PAX5	paired box 5	NM_016734	6.95	1.35E-03
202145_at	LY6E	lymphocyte antigen 6 complex, locus E	NM_001127213	6.83	1.75E-03

203596_s_at	IFIT5	interferon-induced protein with tetratricopeptide repeats 5	NM_012420	6.80	3.84E-04
1552309_a_at	NEXN	nexilin (F actin binding protein)	NM_001172309	6.80	2.34E-03
204994_at	MX2	myxovirus (influenza virus) resistance 2 (mouse)	NM_002463	6.70	6.90E-04
201649_at	UBE2L6	ubiquitin-conjugating enzyme E2L 6	NM_004223	6.66	1.97E-03
206332_s_at	IFI16	interferon, gamma-inducible protein 16	NM_001206567	6.62	2.53E-03
44673_at	SIGLEC1	sialic acid binding Ig-like lectin 1, sialoadhesin	NM_023068	6.54	1.05E-05
228617_at	XAF1	XIAP associated factor 1	NM_017523	6.20	1.76E-04
226103_at	NEXN	nexilin (F actin binding protein)	NM_001172309	6.05	3.38E-04
209761_s_at	SP110	SP110 nuclear body protein	NM_001185015	6.05	9.66E-03
209762_x_at	SP110	SP110 nuclear body protein	NM_001185015	5.95	8.35E-05
213797_at	RSAD2	radical S-adenosyl methionine domain containing 2	NM_080657	5.94	1.97E-03
222793_at	DDX58	DEAD (Asp-Glu-Ala-Asp) box polypeptide 58	NM_014314	5.88	5.31E-04
218986_s_at	DDX60	DEAD (Asp-Glu-Ala-Asp) box polypeptide 60	NM_017631	5.70	4.02E-04
222434_at	ENAH	enabled homolog (Drosophila)	NM_001008493	5.68	4.71E-03
224701_at	PARP14	poly (ADP-ribose) polymerase family, member 14	NM_017554	5.65	9.21E-05
230314_at	---	---	---	5.55	6.38E-03
227807_at	PARP9	poly (ADP-ribose) polymerase family, member 9	NM_001146102	5.48	3.01E-02
208012_x_at	SP110	SP110 nuclear body protein	NM_001185015	5.40	2.41E-04
219209_at	IFIH1	interferon induced with helicase C domain 1	NM_022168	5.39	6.50E-04
208392_x_at	SP110	SP110 nuclear body protein	NM_001185015	5.30	1.45E-03
221044_s_at	TRIM34 /// TRIM6	tripartite motif containing 34 /// TRIM6-TRIM34 readthrough	NM_001003819	5.28	4.27E-03
204698_at	ISG20	interferon stimulated exonuclease gene 20kDa	NM_002201	5.16	1.64E-02
AFFX-HUMISGF3A/ M97935_MA_at	STAT1	signal transducer and activator of transcription 1, 91kDa	NM_007315	5.12	1.67E-03
201141_at	GPNMB	glycoprotein (transmembrane) nmb	NM_001005340	4.97	1.32E-03
210163_at	CXCL11	chemokine (C-X-C motif) ligand 11	NM_005409	4.85	1.79E-02
210797_s_at	OASL	2'-5'-oligoadenylate synthetase-like	NM_001261825	4.84	1.75E-02
AFFX-HUMISGF3A/ M97935_MB_at	STAT1	signal transducer and activator of transcription 1, 91kDa	NM_007315	4.83	9.64E-04
214464_at	CDC42BPA	CDC42 binding protein kinase alpha (DMPK-like)	NM_003607	4.76	2.35E-03
204211_x_at	EIF2AK2	eukaryotic translation initiation factor 2-alpha kinase 2	NM_001135651	4.75	9.37E-06
232155_at	RNF213	ring finger protein 213	NM_001256071	4.63	1.90E-03
33304_at	ISG20	interferon stimulated exonuclease gene 20kDa	NM_002201	4.58	2.03E-03
208894_at	HLA-DRA	major histocompatibility complex, class II, DR alpha	NM_019111	4.55	1.44E-03
218559_s_at	MAFB	v-maf musculoaponeurotic fibrosarcoma oncogene homolog B (avian)	NM_005461	4.54	3.67E-04
238327_at	ODF3B	outer dense fiber of sperm tails 3B	NM_001014440	4.53	1.28E-02
211267_at	HESX1	HESX homeobox 1	NM_003865	4.42	1.79E-05
213294_at	EIF2AK2	eukaryotic translation initiation factor 2-alpha kinase 2	NM_001135651	4.42	8.72E-05
235146_at	TMCC3	transmembrane and coiled-coil domain family 3	NM_020698	4.39	5.66E-03
206553_at	OAS2	2'-5'-oligoadenylate synthetase 2, 69/71kDa	NM_001032731	4.31	1.22E-03
217502_at	IFIT2	interferon-induced protein with tetratricopeptide repeats 2	NM_001547	4.28	1.75E-03
53720_at	C19orf66	chromosome 19 open reading frame 66	NM_018381	4.28	7.23E-04
210657_s_at	Sept4	septin 4	NM_001198713	4.25	4.18E-03
228531_at	SAMD9	sterile alpha motif domain containing 9	NM_001193307	4.16	6.36E-04
219691_at	SAMD9	sterile alpha motif domain containing 9	NM_001193307	4.10	8.45E-04
AFFX-HUMISGF3A/ M97935_5_at	STAT1	signal transducer and activator of transcription 1, 91kDa	NM_007315	4.02	6.57E-03
210705_s_at	TRIM5	tripartite motif containing 5	NM_033034	4.00	3.80E-03
**Downregulated**					
233472_at	TCP11L1	t-complex 11 (mouse)-like 1	NM_001145541	-4.24	1.72E-04
