# Supplementary material for: Antiviral Protection via RdRP-Mediated Stable Activation of Innate Immunity
Source: PLoS Pathog. 2015 Dec 3;11(12):e1005311. doi: 10.1371/journal.ppat.1005311 (PMC4669089; doi:10.1371/journal.ppat.1005311)
Supplement: S5 Table — Differential gene expression in THP-1 cells expressing a codon-optimized RdRP, RdRPΔrna, transgene (n = 2) compared to THP-1 empty vector control cells (n = 2). Gene chip was analyzed as described in methods. Only genes with a fold change in expression >4.0 or <-4.0 and a p-value of <0.05 are shown. References to gene expression made in the body of the paper represent the most upregulated probeset related to that gene. (PDF) [file ppat.1005311.s010.pdf]

**S5 Table. List of genes differentially expressed in codon-optimized RdRP THP-1 cells.** Differential gene expression in THP-1 cells expressing a codon-optimized RdRP, RdRPΔrna, transgene (n=2) compared to THP-1 empty vector control cells (n=2). Gene chip was analyzed as described in methods. Only genes with a fold change in expression >4.0 or <-4.0 and a p-value of <0.05 are shown. References to gene expression made in the body of the paper represent the most upregulated probeset related to that gene.

| Probeset ID | Gene Symbol | Gene Title | RefSeq ID | Fold Change | P-value |
|---|---|---|---|---|---|
| 202411_at | IFI27 | interferon, alpha-inducible protein 27 | NM_001130080 | 474.28 | 2.72E-03 |
| 204439_at | IFI44L | interferon-induced protein 44-like | NM_006820 | 169.36 | 4.62E-04 |
| 213293_s_at | TRIM22 | tripartite motif containing 22 | NM_001199573 | 55.25 | 8.56E-04 |
| 214453_s_at | IFI44 | interferon-induced protein 44 | NM_006417 | 52.37 | 3.04E-04 |
| 202086_at | MX1 | myxovirus (influenza virus) resistance 1 | NM_001144925 | 49.47 | 9.39E-05 |
| 203153_at | IFIT1 | interferon-induced protein with tetratricopeptide repeats 1 | NM_001548 | 29.85 | 3.39E-04 |
| 214022_s_at | IFITM1 | interferon induced transmembrane protein 1 | NM_003641 | 21.32 | 4.69E-04 |
| 227609_at | EPSTI1 | epithelial stromal interaction 1 (breast) | NM_001002264 | 20.14 | 1.33E-04 |
| 201601_x_at | IFITM2 | interferon induced transmembrane protein 2 | NM_006435 | 19.95 | 2.21E-03 |
| 204415_at | IFI6 | interferon, alpha-inducible protein 6 | NM_002038 | 19.92 | 4.87E-05 |
| 204972_at | OAS2 | 2'-5'-oligoadenylate synthetase 2, 69/71kDa | NM_001032731 | 18.19 | 1.86E-04 |
| 235276_at | EPSTI1 | epithelial stromal interaction 1 (breast) | NM_001002264 | 14.80 | 1.27E-03 |
| 202869_at | OAS1 | 2'-5'-oligoadenylate synthetase 1, 40/46kDa | NM_001032409 | 13.75 | 4.99E-03 |
| 205552_s_at | OAS1 | 2'-5'-oligoadenylate synthetase 1, 40/46kDa | NM_001032409 | 13.09 | 1.26E-03 |
| 219519_s_at | SIGLEC1 | sialic acid binding Ig-like lectin 1, sialoadhesin | NM_023068 | 12.16 | 1.31E-02 |
| 226757_at | IFIT2 | interferon-induced protein with tetratricopeptide repeats 2 | NM_001547 | 12.04 | 1.38E-04 |
| 229450_at | IFIT3 | interferon-induced protein with tetratricopeptide repeats 3 | NM_001031683 | 11.92 | 1.20E-04 |
| 202686_s_at | AXL | AXL receptor tyrosine kinase | NM_001699 | 11.55 | 8.70E-04 |
| 208965_s_at | IFI16 | interferon, gamma-inducible protein 16 | NM_001206567 | 11.28 | 5.86E-06 |
| 205483_s_at | ISG15 | ISG15 ubiquitin-like modifier | NM_005101 | 10.49 | 3.87E-04 |
| 203559_s_at | ABP1 | amiloride binding protein 1 (amine oxidase (copper-containing)) | NM_001091 | 8.67 | 4.08E-04 |
| 214464_at | CDC42BPA | CDC42 binding protein kinase alpha (DMPK-like) | NM_003607 | 8.32 | 8.87E-05 |
| 235643_at | SAMD9L | sterile alpha motif domain containing 9-like | NM_152703 | 8.02 | 1.11E-04 |
| 202207_at | ARL4C | ADP-ribosylation factor-like 4C | NM_005737 | 7.92 | 3.33E-03 |
| 206133_at | XAF1 | XIAP associated factor 1 | NM_017523 | 7.78 | 1.72E-03 |
| 211122_s_at | CXCL11 | chemokine (C-X-C motif) ligand 11 | NM_005409 | 7.77 | 1.02E-03 |
| 204533_at | CXCL10 | chemokine (C-X-C motif) ligand 10 | NM_001565 | 7.74 | 5.38E-03 |
| 205041_s_at | ORM1 /// ORM2 | orosomucoid 1 /// orosomucoid 2 | NM_000607 /// NM_000608 | 7.45 | 3.54E-03 |
| 219863_at | HERC5 | HECT and RLD domain containing E3 ubiquitin protein ligase 5 | NM_016323 | 6.91 | 2.10E-04 |
| 200923_at | LGALS3BP | lectin, galactoside-binding, soluble, 3 binding protein | NM_005567 | 6.69 | 3.93E-04 |
| 218400_at | OAS3 | 2'-5'-oligoadenylate synthetase 3, 100kDa | NM_006187 | 6.54 | 3.45E-04 |
| 204747_at | IFIT3 | interferon-induced protein with tetratricopeptide repeats 3 | NM_001031683 | 6.36 | 1.41E-04 |
| 214059_at | IFI44 | Interferon-induced protein 44 | NM_006417 | 6.20 | 1.08E-02 |
| 219211_at | USP18 | ubiquitin specific peptidase 18 | NM_017414 | 6.11 | 5.37E-03 |
| 203595_s_at | IFIT5 | interferon-induced protein with tetratricopeptide repeats 5 | NM_012420 | 6.06 | 8.24E-04 |
| 228607_at | OAS2 | 2'-5'-oligoadenylate synthetase 2, 69/71kDa | NM_001032731 | 5.76 | 5.55E-03 |
| 230036_at | SAMD9L | sterile alpha motif domain containing 9-like | NM_152703 | 5.64 | 5.52E-03 |
| 205660_at | OASL | 2'-5'-oligoadenylate synthetase-like | NM_001261825 | 5.57 | 1.57E-03 |
| 223220_s_at | PARP9 | poly (ADP-ribose) polymerase family, member 9 | NM_001146102 | 5.46 | 6.38E-05 |
| 202206_at | ARL4C | ADP-ribosylation factor-like 4C | NM_005737 | 5.36 | 2.62E-03 |
| 209969_s_at | STAT1 | signal transducer and activator of transcription 1, 91kDa | NM_007315 | 5.34 | 2.01E-05 |
| 212203_x_at | IFITM3 | interferon induced transmembrane protein 3 | NM_021034 | 5.25 | 8.39E-04 |
| 226603_at | SAMD9L | sterile alpha motif domain containing 9-like | NM_152703 | 5.14 | 1.22E-04 |
| 216565_x_at | --- | --- | --- | 4.92 | 8.84E-04 |
| 228617_at | XAF1 | XIAP associated factor 1 | NM_017523 | 4.87 | 4.40E-04 |
| 208966_x_at | IFI16 | interferon, gamma-inducible protein 16 | NM_001206567 | 4.66 | 1.16E-04 |
| 208436_s_at | IRF7 | interferon regulatory factor 7 | NM_001572 | 4.56 | 4.13E-03 |
| 206332_s_at | IFI16 | interferon, gamma-inducible protein 16 | NM_001206567 | 4.45 | 1.97E-03 |
| 220059_at | STAP1 | signal transducing adaptor family member 1 | NM_012108 | 4.37 | 1.17E-03 |

| | | | | | |
|---|---|---|---|---|---|
| 226702_at | CMPK2 | cytidine monophosphate (UMP-CMP) kinase 2, mitochondrial | NM_001256477 | 4.37 | 2.56E-03 |
| 242625_at | RSAD2 | radical S-adenosyl methionine domain containing 2 | NM_080657 | 4.37 | 1.21E-03 |
| 219352_at | HERC6 | HECT and RLD domain containing E3 ubiquitin protein ligase 6 | NM_001013000 | 4.36 | 1.75E-04 |
| 223980_s_at | SP110 | SP110 nuclear body protein | NM_001185015 | 4.28 | 7.65E-04 |
| 204994_at | MX2 | myxovirus (influenza virus) resistance 2 (mouse) | NM_002463 | 4.26 | 1.21E-03 |
| 218986_s_at | DDX60 | DEAD (Asp-Glu-Ala-Asp) box polypeptide 60 | NM_017631 | 4.15 | 1.26E-03 |
| 202145_at | LY6E | lymphocyte antigen 6 complex, locus E | NM_001127213 | 4.12 | 3.45E-03 |
| 44673_at | SIGLEC1 | sialic acid binding Ig-like lectin 1, sialoadhesin | NM_023068 | 4.06 | 2.13E-03 |
| 218943_s_at | DDX58 | DEAD (Asp-Glu-Ala-Asp) box polypeptide 58 | NM_014314 | 4.05 | 1.84E-03 |
| 209761_s_at | SP110 | SP110 nuclear body protein | NM_001185015 | 4.05 | 1.67E-02 |
| 210512_s_at | VEGFA | vascular endothelial growth factor A | NM_001025366 | 4.02 | 1.42E-03 |
| **Downregulated** | | | | | |
| 239710_at | FIGN | fidgetin | NM_018086 | -4.63 | 2.14E-03 |
| 233472_at | TCP11L1 | t-complex 11 (mouse)-like 1 | NM_001145541 | -4.84 | 2.44E-03 |
