# Supplementary material for: Antiviral Protection via RdRP-Mediated Stable Activation of Innate Immunity
Source: PLoS Pathog. 2015 Dec 3;11(12):e1005311. doi: 10.1371/journal.ppat.1005311 (PMC4669089; doi:10.1371/journal.ppat.1005311)
Supplement: S6 Table — Murine genes upregulated (>4-fold) in tissues of uninfected RdRP mice and human genes upregulated (>4-fold) in RdRP-expressing THP-1 monocytes encode proteins with prominent roles in broad-spectrum viral attenuation. (PDF) [file ppat.1005311.s011.pdf]

Antiviral effector	Murine genes	Human genes	Summary of antiviral effector functions
**2'-5' OAS**	***Oas1,<br>Oasl2***	***OAS1,<br>OAS2,<br>OAS3,<br>OASL***	OAS enzymes bind dsRNA promoting conversion of ATP to 2'-5'-linked oligoadenlates (2-5A) [46]. 2-5A is an unique ligand that binds to and activates latent RNase L which nonspecifically binds and degrades RNA. Mechanism of *hOasL* antiviral activity unknown; broadly antiviral effects against EMCV [47] and hepatitis C virus [36].
**IFIT**	***Ifit1,<br>Ifit2,<br>Ifit3***	***IFIT1,<br>IFIT2,<br>IFIT3,<br>IFIT5***	IFIT1 binds 5'-triphosphate RNA which mediates formation of a large protein complex containing IFIT2 and IFIT3. This IFIT complex inhibits translation by sequestering viral nucleic acid [48,49].
**RIG-I-like receptors**	***Ddx58/Rig-I,<br>Ifih1/Mda5***	***DDX58/RIG-I,<br>IFIH1/MDA5***	Pathogen Recognition Receptors (PRRs); RNA helicases with vital roles in cytosolic viral RNA sensing and activation of the innate immune antiviral response [21]. RIG-I and MDA5 identified as broadly acting effectors against hepatitis C, West Nile, and Venezuelan equine encephalitis viruses [36].
**PKR**	***Eif2ak2***	***EIF2AK2***	PKR binds viral nucleic acid (dsRNA, 5'-triphosphate RNA) which induces its activation [50]. Phosphorylation of eIF2α by PKR leads to inhibition of viral replication through arrest of global cellular translation. PKR also inhibits cytoskeletal rearrangement required for actin-dependent viral entry [51].
**Myxovirus resistance**	***Mx1,<br>Mx2***	***MX1,<br>MX2***	Dynamin-like GTPases; Mx1/MxA oligomerized to trap viral elements (nucleocapsids, mRNA) prior to genome amplification [52,53]. Mx2/MxB inactivates post-entry HIV-1 reverse transcription complexes and inhibits viral cDNA nuclear accumulation [54].
**IFITM**	***Ifitm1,<br>Ifitm2,<br>Ifitm3***	***IFITM1,<br>IFITM2,<br>IFITM3***	Restrict early stage influenza A, West Nile, and Dengue virus replication [55]. Ifitm3 inhibits influenza A virus cytosolic entry from the late endosome [56] and is critical for intrinsic resistance *in vivo* [57].
**Virus inhibitory protein**	***Rsad2/Viperin***	***RSAD2/ VIPERIN***	Viperin can disrupt transport of essential viral structural peptides within the secretory pathway [58] and perturb lipid-raft-mediated influenza release from the plasma membrane [59].
**IFI27**	***Ifi27l2a***	***IFI27***	Mechanism unknown. Reduced murine susceptibility to fatal viral encephalitis with Sindbis virus attributed to increased expression of *Ifi27l2a* [60]. Broadly antiviral effects in primary cortical neurons against St. Louis encephalitis, West Nile, and mouse hepatitis viruses [38].
**ISG15**	***Isg15***	***ISG15***	Blocks ubiquitination of Gag and inhibits release of HIV-1 virions [61]. ISG15 attachment imposes a dominant-negative structural effect on targeted proteins. In virally infected cells, increased ISG15 expression promotes global ISG15-conjugation in an attempt to modify viral proteins [62].

S6 Table. RdRP expression triggers an innate antiviral response in uninfected hosts. Murine genes upregulated (> 4-fold) in tissues of uninfected RdRP mice and human genes upregulated (>4-fold) in RdRP-expressing THP-1 monocytes encode proteins with prominent roles in broad-spectrum viral attenuation.
