# Supplementary material for: Antiviral Protection via RdRP-Mediated Stable Activation of Innate Immunity
Source: PLoS Pathog. 2015 Dec 3;11(12):e1005311. doi: 10.1371/journal.ppat.1005311 (PMC4669089; doi:10.1371/journal.ppat.1005311)
Supplement: S7 Table — Differential gene expression in THP-1 cells expressing the catalytic mutant, RdRPΔcat, transgene (n = 2) compared to THP-1 empty vector control cells (n = 2). Gene chip was analyzed as described in methods. Only genes with a fold change in expression >4.0 or <-4.0 and a p-value of <0.05 are shown. References to gene expression made in the body of the paper represent the most upregulated probeset related to that gene. (PDF) [file ppat.1005311.s012.pdf]

S7 Table. List of genes differentially expressed in RdRPΔcat THP-1 cells. Differential gene expression in THP-1 cells expressing the catalytic mutant, RdRPΔcat, transgene (n=2) compared to THP-1 empty vector control cells (n=2). Gene chip was analyzed as described in methods. Only genes with a fold change in expression >4.0 or <-4.0 and a p-value of <0.05 are shown. References to gene expression made in the body of the paper represent the most upregulated probeset related to that gene.

Probeset ID	Gene Symbol	Gene Title	RefSeq ID	Fold Chang	P-value
205041_s_at	ORM1 ///	orosomucoid 1 ///	NM_000607 ///	154.14	1.82E-05
	ORM2	orosomucoid 2	NM_000608		
204351_at	S100P	S100 calcium binding protein P	NM_005980	69.43	6.87E-04
205040_at	ORM1	orosomucoid 1	NM_000607	56.55	8.38E-05
210587_at	INHBE	inhibin, beta E	NM_031479	45.99	3.81E-03
210512_s_at	VEGFA	vascular endothelial growth factor A	NM_001025366	40.15	3.76E-04
209230_s_at	NUPR1	nuclear protein, transcriptional regulator, 1	NM_001042483	29.96	8.01E-04
235334_at	ST6GALNAC3	ST6	NM_001160011	27.02	1.71E-03
212912_at	RPS6KA2	ribosomal protein S6 kinase, 90kDa, polypeptide 2	NM_001006932	25.33	6.02E-03
202887_s_at	DDIT4	DNA-damage-inducible transcript 4	NM_019058	24.81	7.78E-05
232689_at	LOC284561	uncharacterized LOC284561	XR_110828	19.92	7.07E-04
205047_s_at	ASNS	asparagine synthetase (glutamine-hydrolyzing)	NM_001178075	19.30	6.50E-06
225806_at	AJUBA	ajuba LIM protein	NM_032876	16.74	7.80E-04
230741_at	---	---	---	15.68	4.14E-04
233737_s_at	LOC284561	uncharacterized LOC284561	XR_110828	14.89	7.35E-03
225846_at	ESRP1	epithelial splicing regulatory protein 1	NM_001034915	14.82	6.94E-04
1553764_a_at	AJUBA	ajuba LIM protein	NM_032876	14.49	2.07E-05
212486_s_at	FYN	FYN oncogene related to SRC, FGR, YES	NM_001242779	13.49	1.68E-03
204999_s_at	ATF5	activating transcription factor 5	NM_001193646	13.42	2.45E-04
204998_s_at	ATF5	activating transcription factor 5	NM_001193646	11.91	6.18E-04
236407_at	KCNE1	potassium voltage-gated channel, Isk-related family, member 1	NM_000219	11.43	8.09E-03
232000_at	---	---	---	11.23	8.49E-04
219892_at	TM6SF1	transmembrane 6 superfamily member 1	NM_001144903	11.21	1.36E-02
220892_s_at	PSAT1	phosphoserine aminotransferase 1	NM_021154	10.35	3.54E-04
207302_at	SGCG	sarcoglycan, gamma (35kDa dystrophin-associated glycoprotein)	NM_000231	10.32	2.88E-03
229288_at	EPHA7	EPH receptor A7	NM_004440	10.27	9.71E-03
212233_at	MAP1B	microtubule-associated protein 1B	NM_005909	9.69	1.83E-03
221577_x_at	GDF15	growth differentiation factor 15	NM_004864	9.60	1.61E-02
228141_at	GPX8	glutathione peroxidase 8 (putative)	NM_001008397	9.45	9.42E-03
210105_s_at	FYN	FYN oncogene related to SRC, FGR, YES	NM_001242779	9.16	1.45E-03
218145_at	TRIB3	tribbles homolog 3 (Drosophila)	NM_021158	9.15	1.16E-05
231202_at	ALDH1L2	aldehyde dehydrogenase 1 family, member L2	NM_001034173	8.89	7.96E-04
219519_s_at	SIGLEC1	sialic acid binding Ig-like lectin 1, sialoadhesin	NM_023068	8.87	1.79E-02
213293_s_at	TRIM22	tripartite motif containing 22	NM_001199573	8.81	3.08E-03
223196_s_at	SESN2	sestrin 2	NM_031459	8.60	1.08E-02
222668_at	KCTD15	potassium channel tetramerisation domain containing 15	NM_001129994	8.40	1.86E-03
205569_at	LAMP3	lysosomal-associated membrane protein 3	NM_014398	8.39	3.79E-04
202686_s_at	AXL	AXL receptor tyrosine kinase	NM_001699	8.38	2.07E-04
223062_s_at	PSAT1	phosphoserine aminotransferase 1	NM_021154	8.09	9.13E-05
243483_at	TRPM8	transient receptor potential cation channel, subfamily M, member 8	NM_024080	7.97	1.16E-03
219121_s_at	ESRP1	epithelial splicing regulatory protein 1	NM_001034915	7.91	2.06E-03
226084_at	MAP1B	microtubule-associated protein 1B	NM_005909	7.89	8.74E-05
217127_at	CTH	cystathionase (cystathionine gamma-lyase)	NM_001190463	7.77	1.06E-03
239675_at	LOC283143	uncharacterized LOC283143	NR_034148	7.61	3.99E-03
222156_x_at	CCPG1 ///	cell cycle progression 1 ///	NM_001204450 ///	7.47	5.40E-05
	DYX1C1-CCPG1	DYX1C1-CCPG1 readthrough (non-protein coding)	NR_037923		
204415_at	IFI6	interferon, alpha-inducible protein 6	NM_002038	7.31	1.66E-04
214022_s_at	IFITM1	interferon induced transmembrane protein 1	NM_003641	6.98	2.06E-04
221511_x_at	CCPG1 ///	cell cycle progression 1 ///	NM_001204450 ///	6.82	1.04E-03
	DYX1C1-CCPG1	DYX1C1-CCPG1 readthrough (non-protein coding)	NR_037923		

213332_at	PAPPA2	pappalysin 2	NM_020318	6.78	7.22E-03
223195_s_at	SESN2	sestrin 2	NM_031459	6.73	1.44E-02
203438_at	STC2	stanniocalcin 2	NM_003714	6.72	2.73E-04
225807_at	AJUBA	ajuba LIM protein	NM_032876	6.65	1.71E-03
201397_at	PHGDH	phosphoglycerate dehydrogenase	NM_006623	6.63	4.87E-04
200629_at	WARS	tryptophanyl-tRNA synthetase	NM_004184	6.53	1.53E-03
203961_at	NEBL	nebulette	NM_001173484	6.17	1.24E-03
1554503_a_at	OSCAR	osteoclast associated, immunoglobulin-like receptor	NM_130771	6.16	3.95E-03
242477_at	TTC39B	tetratricopeptide repeat domain 39B	NM_001168339	6.11	4.05E-03
238635_at	C5orf28	chromosome 5 open reading frame 28	NM_022483	6.09	2.31E-03
204439_at	IFI44L	interferon-induced protein 44-like	NM_006820	6.07	3.74E-04
201195_s_at	SLC7A5	solute carrier family 7, member 5	NM_003486	6.00	2.64E-04
210002_at	GATA6	GATA binding protein 6	NM_005257	5.92	1.63E-03
203962_s_at	NEBL	nebulette	NM_001173484	5.91	1.11E-03
214464_at	CDC42BPA	CDC42 binding protein kinase alpha (DMPK-like)	NM_003607	5.84	1.23E-02
213524_s_at	G0S2	G0/G1switch 2	NM_015714	5.83	8.49E-04
204284_at	PPP1R3C	protein phosphatase 1, regulatory subunit 3C	NM_005398	5.77	7.01E-04
236236_at	WNK3	WNK lysine deficient protein kinase 3	NM_001002838	5.74	1.32E-03
201601_x_at	IFITM2	interferon induced transmembrane protein 2	NM_006435	5.72	1.05E-03
201141_at	GPNMB	glycoprotein (transmembrane) nmb	NM_001005340	5.66	9.45E-04
214152_at	CCPG1 /// DYX1C1-CCPG1	cell cycle progression 1 /// DYX1C1-CCPG1 readthrough (non-protein coding)	NM_001204450 /// NR_037923	5.62	1.14E-03
209525_at	HDGFRP3	Hepatoma-derived growth factor, related protein 3	NM_016073	5.62	1.36E-03
227628_at	GPX8	glutathione peroxidase 8 (putative)	NM_001008397	5.62	2.57E-03
201008_s_at	TXNIP	thioredoxin interacting protein	NM_006472	5.56	8.77E-04
207076_s_at	ASS1	argininosuccinate synthase 1	NM_000050	5.55	1.48E-04
226267_at	JDP2	Jun dimerization protein 2	NM_001135047	5.39	1.26E-04
211962_s_at	ZFP36L1	zinc finger protein 36, C3H type-like 1	NM_001244698	5.38	7.05E-03
202847_at	PCK2	phosphoenolpyruvate carboxykinase 2 (mitochondrial)	NM_001018073	5.28	1.39E-04
243541_at	IL31RA	interleukin 31 receptor A	NM_001242636	5.22	6.23E-05
225864_at	FAM84B	family with sequence similarity 84, member B	NM_174911	5.17	4.89E-04
203153_at	IFIT1	interferon-induced protein with tetratricopeptide repeats 1	NM_001548	5.17	6.10E-03
207996_s_at	C18orf1	chromosome 18 open reading frame 1	NM_001003674	5.14	3.04E-03
204174_at	ALOX5AP	arachidonate 5-lipoxygenase-activating protein	NM_001204406	5.12	7.48E-05
201009_s_at	TXNIP	thioredoxin interacting protein	NM_006472	5.09	1.74E-03
232267_at	GPR133	G protein-coupled receptor 133	NM_198827	5.08	5.91E-05
202086_at	MX1	myxovirus (influenza virus) resistance 1	NM_001144925	5.05	1.19E-04
200628_s_at	WARS	tryptophanyl-tRNA synthetase	NM_004184	5.03	3.73E-04
213158_at	---	---	---	5.00	4.43E-03
216033_s_at	FYN	FYN oncogene related to SRC, FGR, YES	NM_001242779	4.96	2.27E-03
234976_x_at	MTHFD2	methylenetetrahydrofolate dehydrogenase (NADP+ dependent) 2	NM_001040409	4.94	3.30E-03
235191_at	---	---	---	4.91	1.37E-03
202869_at	OAS1	2'-5'-oligoadenylate synthetase 1, 40/46kDa	NM_001032409	4.90	1.72E-02
203562_at	FEZ1	fasciculation and elongation protein zeta 1 (zygin I)	NM_005103	4.88	2.07E-04
214151_s_at	CCPG1 /// DYX1C1-CCPG1	cell cycle progression 1 /// DYX1C1-CCPG1 readthrough (non-protein coding)	NM_001204450 /// NR_037923	4.88	5.70E-04
1555788_a_at	TRIB3	tribbles homolog 3 (Drosophila)	NM_021158	4.87	2.66E-03
219270_at	CHAC1	ChaC, cation transport regulator homolog 1 (E. coli)	NM_001142776	4.84	5.28E-02
209409_at	GRB10	growth factor receptor-bound protein 10	NM_001001549	4.83	1.07E-04
204961_s_at	NCF1	neutrophil cytosolic factor 1	NM_000265	4.83	9.20E-04
204953_at	SNAP91	synaptosomal-associated protein, 91kDa homolog (mouse)	NM_001242792	4.83	2.28E-04
202529_at	PRPSAP1	phosphoribosyl pyrophosphate synthetase-associated protein 1	NM_002766	4.77	5.42E-05
201645_at	TNC	tenascin C	NM_002160	4.75	8.81E-03
214453_s_at	IFI44	interferon-induced protein 44	NM_006417	4.75	1.87E-03
221156_x_at	CCPG1	cell cycle progression 1	NM_001204450	4.72	3.05E-03

205552_s_at	OAS1	2'-5'-oligoadenylate synthetase 1, 40/46kDa	NM_001032409	4.72	1.82E-03
222664_at	KCTD15	potassium channel tetramerisation domain containing 15	NM_001129994	4.66	1.82E-02
218332_at	BEX1	brain expressed, X-linked 1	NM_018476	4.66	1.26E-03
238533_at	EPHA7	EPH receptor A7	NM_004440	4.66	2.66E-02
208018_s_at	HCK	hemopoietic cell kinase	NM_001172129	4.64	1.17E-05
204446_s_at	ALOX5	arachidonate 5-lipoxygenase	NM_000698	4.64	2.26E-03
224559_at	MALAT1	metastasis associated lung adenocarcinoma transcript 1	NR_002819	4.63	7.81E-05
226181_at	TUBE1	tubulin, epsilon 1	NM_016262	4.61	2.83E-04
232282_at	WNK3	WNK lysine deficient protein kinase 3	NM_001002838	4.60	1.21E-02
206380_s_at	CFP	complement factor properdin	NM_001145252	4.59	6.19E-05
212187_x_at	PTGDS	prostaglandin D2 synthase 21kDa (brain)	NM_000954	4.58	2.36E-04
209369_at	ANXA3	annexin A3	NM_005139	4.54	7.23E-03
235459_at	RNF41	ring finger protein 41	NM_001242826	4.51	2.28E-03
235004_at	RBM24	RNA binding motif protein 24	NM_001143941	4.49	2.62E-03
242404_at	---	---	---	4.46	2.36E-04
211748_x_at	PTGDS	prostaglandin D2 synthase 21kDa (brain)	NM_000954	4.44	1.48E-02
228766_at	CD36	CD36 molecule (thrombospondin receptor)	NM_000072	4.40	3.40E-03
227265_at	FGL2	fibrinogen-like 2	NM_006682	4.40	3.04E-02
209031_at	CADM1	cell adhesion molecule 1	NM_001098517	4.38	9.32E-03
205054_at	NEB	nebulin	NM_001164507	4.33	8.84E-04
206519_x_at	SIGLEC6	sialic acid binding Ig-like lectin 6	NM_001177547	4.30	5.92E-03
208763_s_at	TSC22D3	TSC22 domain family, member 3	NM_001015881	4.28	5.08E-05
206085_s_at	CTH	cystathionase (cystathionine gamma-lyase)	NM_001190463	4.26	7.49E-03
201150_s_at	TIMP3	TIMP metallopeptidase inhibitor 3	NM_000362	4.25	6.17E-04
229695_at	---	---	---	4.22	2.53E-03
203695_s_at	DFNA5	deafness, autosomal dominant 5	NM_001127453	4.18	6.98E-03
221865_at	C9orf91	chromosome 9 open reading frame 91	NM_153045	4.14	1.93E-03
241869_at	APOL6	apolipoprotein L, 6	NM_030641	4.14	4.16E-03
232297_at	KLHL5	kelch-like 5 (Drosophila)	NM_001007075	4.13	2.37E-03
207299_s_at	GRM1	glutamate receptor, metabotropic 1	NM_000838	4.11	1.83E-03
210095_s_at	IGFBP3	insulin-like growth factor binding protein 3	NM_000598	4.10	3.43E-02
219716_at	APOL6	apolipoprotein L, 6	NM_030641	4.08	5.25E-04
221538_s_at	PLXNA1	plexin A1	NM_032242	4.06	8.32E-04
200924_s_at	SLC3A2	solute carrier family 3	NM_001012661	4.04	4.77E-05
227755_at	---	---	---	4.03	8.69E-04
228933_at	NHS	Nance-Horan syndrome (congenital cataracts and dental anomalies)	NM_001136024	4.02	1.54E-03
**Downregulated**					
220840_s_at	C1orf112	chromosome 1 open reading frame 112	NM_018186	-4.01	1.04E-04
205114_s_at	CCL3	chemokine (C-C motif) ligand 3	NM_001001437	-4.02	2.01E-03
236976_at	FANCA	Fanconi anemia, complementation group A	NM_000135	-4.02	4.59E-03
222680_s_at	DTL	denticleless E3 ubiquitin protein ligase homolog (Drosophila)	NM_016448	-4.02	7.21E-05
228774_at	CEP78	centrosomal protein 78kDa	NM_001098802	-4.03	1.80E-03
211506_s_at	IL8	interleukin 8	NM_000584	-4.06	3.39E-02
220085_at	HELLS	helicase, lymphoid-specific	NM_018063	-4.06	3.84E-03
203625_x_at	SKP2	S-phase kinase-associated protein 2, E3 ubiquitin protein ligase	NM_001243120	-4.07	2.95E-05
212949_at	NCAPH	non-SMC condensin I complex, subunit H	NM_015341	-4.07	3.83E-03
202085_at	TJP2	tight junction protein 2 (zona occludens 2)	NM_001170414	-4.09	4.43E-04
1562484_at	C17orf104	chromosome 17 open reading frame 104	NM_001033659	-4.10	7.70E-04
205394_at	CHEK1	checkpoint kinase 1	NM_001114121	-4.10	1.98E-03
210387_at	HIST1H2BC	histone cluster 1, H2bc	NM_003518	-4.11	1.80E-02
239253_at	---	---	---	-4.11	2.50E-03
205085_at	ORC1	origin recognition complex, subunit 1	NM_001190818	-4.13	1.85E-03
222036_s_at	MCM4	minichromosome maintenance complex component 4	NM_005914	-4.13	3.08E-03
235609_at	BRIP1	BRCA1 interacting protein C-terminal helicase 1	NM_032043	-4.15	1.34E-03
204767_s_at	FEN1	flap structure-specific endonuclease 1	NM_004111	-4.21	1.08E-04

202589_at	TYMS	thymidylate synthetase	NM_001071	-4.24	1.65E-04
212021_s_at	MKI67	antigen identified by monoclonal antibody Ki-67	NM_001145966	-4.26	1.06E-03
202107_s_at	MCM2	minichromosome maintenance complex component 2	NM_004526	-4.26	5.64E-04
229551_x_at	ZNF367	zinc finger protein 367	NM_153695	-4.30	5.11E-04
204768_s_at	FEN1	flap structure-specific endonuclease 1	NM_004111	-4.31	4.34E-03
204033_at	TRIP13	thyroid hormone receptor interactor 13	NM_001166260	-4.32	1.14E-03
226278_at	SVIP	small VCP/p97-interacting protein	NM_148893	-4.36	2.41E-03
204728_s_at	WDHD1	WD repeat and HMG-box DNA binding protein 1	NM_001008396	-4.37	6.96E-03
204603_at	EXO1	exonuclease 1	NM_003686	-4.37	1.52E-03
203022_at	RNASEH2A	ribonuclease H2, subunit A	NM_006397	-4.38	7.24E-06
209891_at	SPC25	SPC25, NDC80 kinetochore complex component, homolog (S. cerevisiae)	NM_020675	-4.38	3.25E-04
215143_at	DPY19L2P2	dpy-19-like 2 pseudogene 2 (C. elegans)	NM_182634	-4.41	1.69E-02
213302_at	PFAS	phosphoribosylformylglycinamidine synthase	NM_012393	-4.44	8.14E-04
39248_at	AQP3	aquaporin 3 (Gill blood group)	NM_004925	-4.44	2.67E-04
204126_s_at	CDC45	cell division cycle 45 homolog (S. cerevisiae)	NM_001178010	-4.46	2.74E-04
204558_at	RAD54L	RAD54-like (S. cerevisiae)	NM_001142548	-4.47	9.13E-04
205419_at	GPR183	G protein-coupled receptor 183	NM_004951	-4.52	9.48E-04
212022_s_at	MKI67	antigen identified by monoclonal antibody Ki-67	NM_001145966	-4.52	2.03E-04
210397_at	DEFB1	defensin, beta 1	NM_005218	-4.53	2.98E-04
235683_at	SESN3	sestrin 3	NM_144665	-4.58	3.52E-03
207865_s_at	BMP8B	bone morphogenetic protein 8b	NM_001720	-4.62	1.95E-03
233472_at	TCP11L1	t-complex 11 (mouse)-like 1	NM_001145541	-4.63	2.36E-03
218723_s_at	RGCC	regulator of cell cycle	NM_014059	-4.65	2.66E-04
205393_s_at	CHEK1	checkpoint kinase 1	NM_001114121	-4.72	1.11E-03
219000_s_at	DSCC1	defective in sister chromatid cohesion 1 homolog (S. cerevisiae)	NM_024094	-4.74	1.21E-03
1554696_s_at	TYMS	thymidylate synthetase	NM_001071	-4.76	5.44E-04
228281_at	C11orf82	chromosome 11 open reading frame 82	NM_145018	-4.79	4.29E-04
207746_at	POLQ	polymerase (DNA directed), theta	NM_199420	-4.81	4.24E-03
216228_s_at	WDHD1	WD repeat and HMG-box DNA binding protein 1	NM_001008396	-4.88	4.94E-03
205909_at	POLE2	polymerase (DNA directed), epsilon 2, accessory subunit	NM_001197330	-4.90	9.19E-04
227350_at	HELLS	helicase, lymphoid-specific	NM_018063	-4.96	1.31E-03
203549_s_at	LPL	lipoprotein lipase	NM_000237	-5.07	3.75E-03
214370_at	S100A8	S100 calcium binding protein A8	NM_002964	-5.09	5.75E-03
212141_at	MCM4	minichromosome maintenance complex component 4	NM_005914	-5.16	5.29E-04
203065_s_at	CAV1	caveolin 1, caveolae protein, 22kDa	NM_001172895	-5.17	5.02E-04
242890_at	---	---	---	-5.19	4.56E-03
205034_at	CCNE2	cyclin E2	NM_004702	-5.34	1.93E-03
211814_s_at	CCNE2	cyclin E2	NM_004702	-5.36	7.63E-03
214240_at	GAL	galanin prepropeptide	NM_015973	-5.44	8.85E-03
206102_at	GINS1	GINS complex subunit 1 (Psf1 homolog)	NM_021067	-5.45	2.87E-04
207269_at	DEFA4	defensin, alpha 4, corticostatin	NM_001925	-5.51	4.47E-03
204695_at	CDC25A	cell division cycle 25 homolog A (S. pombe)	NM_001789	-5.59	2.20E-04
228033_at	E2F7	E2F transcription factor 7	NM_203394	-5.64	3.01E-03
219990_at	E2F8	E2F transcription factor 8	NM_001256371	-5.67	5.96E-05
222037_at	MCM4	minichromosome maintenance complex component 4	NM_005914	-5.74	3.82E-04
1561079_at	ANKRD28	ankyrin repeat domain 28	NM_001195098	-5.77	1.29E-03
201890_at	RRM2	ribonucleotide reductase M2	NM_001034	-5.79	2.35E-04
221521_s_at	GINS2	GINS complex subunit 2 (Psf2 homolog)	NM_016095	-5.83	1.81E-04
212097_at	CAV1	caveolin 1, caveolae protein, 22kDa	NM_001172895	-5.85	2.32E-04
222162_s_at	ADAMTS1	ADAM metallopeptidase with thrombospondin type 1 motif, 1	NM_006988	-5.86	2.59E-03
223570_at	MCM10	minichromosome maintenance complex component 10	NM_018518	-5.98	2.38E-04
223556_at	HELLS	helicase, lymphoid-specific	NM_018063	-6.00	1.88E-03
1555772_a_at	CDC25A	cell division cycle 25 homolog A (S. pombe)	NM_001789	-6.15	1.47E-02
207725_at	POU4F2	POU class 4 homeobox 2	NM_004575	-6.18	1.71E-04
203535_at	S100A9	S100 calcium binding protein A9	NM_002965	-6.20	1.41E-03

202345_s_at	FABP5	fatty acid binding protein 5 (psoriasis-associated)	NM_001444	-6.34	4.23E-05
222962_s_at	MCM10	minichromosome maintenance complex component 10	NM_018518	-6.48	9.11E-04
243840_at	CLSPN	claspin	NM_001190481	-6.50	1.28E-03
232760_at	TEX15	testis expressed 15	NM_031271	-6.52	1.77E-02
219295_s_at	PCOLCE2	procollagen C-endopeptidase enhancer 2	NM_013363	-6.64	7.65E-04
210567_s_at	SKP2	S-phase kinase-associated protein 2, E3 ubiquitin protein ligase	NM_001243120	-6.88	6.03E-03
202859_x_at	IL8	interleukin 8	NM_000584	-7.32	9.64E-04
203548_s_at	LPL	lipoprotein lipase	NM_000237	-7.32	1.51E-03
205347_s_at	TMSB15A ///	thymosin beta 15a ///	NM_021992	-7.38	1.26E-03
	TMSB15B	thymosin beta 15B	NM_194324		
209773_s_at	RRM2	ribonucleotide reductase M2	NM_001034	-8.13	9.75E-04
220651_s_at	MCM10	minichromosome maintenance complex component 10	NM_018518	-9.15	1.45E-04
206115_at	EGR3	early growth response 3	NM_001199880	-9.16	1.19E-04
202917_s_at	S100A8	S100 calcium binding protein A8	NM_002964	-9.42	3.27E-04
224428_s_at	CDCA7	cell division cycle associated 7	NM_031942	-9.52	9.56E-05
205404_at	HSD11B1	hydroxysteroid (11-beta) dehydrogenase 1	NM_001206741	-15.69	2.56E-04
