# Supplementary material for: Antiviral Protection via RdRP-Mediated Stable Activation of Innate Immunity
Source: PLoS Pathog. 2015 Dec 3;11(12):e1005311. doi: 10.1371/journal.ppat.1005311 (PMC4669089; doi:10.1371/journal.ppat.1005311)
Supplement: S1 References — (DOCX) [file ppat.1005311.s013.docx]

**Supporting References**

46. Justesen J, Hartmann R, Kjeldgaard NO (2000) Gene structure and function of the 2'-5'-oligoadenylate synthetase family. Cellular and molecular life sciences : CMLS 57: 1593-1612.

47. Marques J, Anwar J, Eskildsen-Larsen S, Rebouillat D, Paludan SR, et al. (2008) The p59 oligoadenylate synthetase-like protein possesses antiviral activity that requires the C-terminal ubiquitin-like domain. The Journal of general virology 89: 2767-2772.

48. Pichlmair A, Lassnig C, Eberle CA, Gorna MW, Baumann CL, et al. (2011) IFIT1 is an antiviral protein that recognizes 5'-triphosphate RNA. Nature immunology 12: 624-630.

49. Daffis S, Szretter KJ, Schriewer J, Li J, Youn S, et al. (2010) 2'-O methylation of the viral mRNA cap evades host restriction by IFIT family members. Nature 468: 452-456.

50. Meurs E, Chong K, Galabru J, Thomas NS, Kerr IM, et al. (1990) Molecular cloning and characterization of the human double-stranded RNA-activated protein kinase induced by interferon. Cell 62: 379-390.

51. Irving AT, Wang D, Vasilevski O, Latchoumanin O, Kozer N, et al. (2012) Regulation of actin dynamics by protein kinase R control of gelsolin enforces basal innate immune defense. Immunity 36: 795-806.

52. Gao S, von der Malsburg A, Dick A, Faelber K, Schroder GF, et al. (2011) Structure of myxovirus resistance protein a reveals intra- and intermolecular domain interactions required for the antiviral function. Immunity 35: 514-525.

53. Mitchell PS, Patzina C, Emerman M, Haller O, Malik HS, et al. (2012) Evolution-guided identification of antiviral specificity determinants in the broadly acting interferon-induced innate immunity factor MxA. Cell host & microbe 12: 598-604.

54. Goujon C, Moncorge O, Bauby H, Doyle T, Ward CC, et al. (2013) Human MX2 is an interferon-induced post-entry inhibitor of HIV-1 infection. Nature 502: 559-562.

55. Brass AL, Huang IC, Benita Y, John SP, Krishnan MN, et al. (2009) The IFITM proteins mediate cellular resistance to influenza A H1N1 virus, West Nile virus, and dengue virus. Cell 139: 1243-1254.

56. Feeley EM, Sims JS, John SP, Chin CR, Pertel T, et al. (2011) IFITM3 inhibits influenza A virus infection by preventing cytosolic entry. PLoS pathogens 7: e1002337.

57. Everitt AR, Clare S, Pertel T, John SP, Wash RS, et al. (2012) IFITM3 restricts the morbidity and mortality associated with influenza. Nature 484: 519-523.

58. Chin KC, Cresswell P (2001) Viperin (cig5), an IFN-inducible antiviral protein directly induced by human cytomegalovirus. Proceedings of the National Academy of Sciences of the United States of America 98: 15125-15130.

59. Wang X, Hinson ER, Cresswell P (2007) The interferon-inducible protein viperin inhibits influenza virus release by perturbing lipid rafts. Cell host & microbe 2: 96-105.

60. Labrada L, Liang XH, Zheng W, Johnston C, Levine B (2002) Age-dependent resistance to lethal alphavirus encephalitis in mice: analysis of gene expression in the central nervous system and identification of a novel interferon-inducible protective gene, mouse ISG12. Journal of Virology 76: 11688-11703.

61. Okumura A, Lu G, Pitha-Rowe I, Pitha PM (2006) Innate antiviral response targets HIV-1 release by the induction of ubiquitin-like protein ISG15. Proceedings of the National Academy of Sciences of the United States of America 103: 1440-1445.

62. Durfee LA, Lyon N, Seo K, Huibregtse JM (2010) The ISG15 conjugation system broadly targets newly synthesized proteins: implications for the antiviral function of ISG15. Molecular cell 38: 722-732.
